# The Transcription Factor Foxg1 Promotes Optic Fissure Closure in the Mouse by Suppressing Wnt8b in the Nasal Optic Stalk

**DOI:** 10.1523/JNEUROSCI.0286-17.2017

**Published:** 2017-08-16

**Authors:** Rowena Smith, Yu-Ting Huang, Tian Tian, Dominika Vojtasova, Oscar Mesalles-Naranjo, Steven M. Pollard, Thomas Pratt, David J. Price, Vassiliki Fotaki

**Affiliations:** ^1^Edinburgh Medical School, Biomedical Sciences, Centre for Integrative Physiology, Edinburgh, EH8 9XD, United Kingdom,; ^2^Information Service Division, NHS National Services Scotland, Edinburgh, EH12 9EB, United Kingdom,; ^3^Medical Research Council Centre for Regenerative Medicine, Edinburgh, EH16 4UU, United Kingdom, and; ^4^Edinburgh Cancer Research UK Cancer Centre, Edinburgh, EH16 4UU, United Kingdom

**Keywords:** Coloboma, Foxg1, mouse, optic cup, optic fissure, Wnt8b

## Abstract

During vertebrate eye morphogenesis, a transient fissure forms at its inferior part, known as the optic fissure. This will gradually close, giving rise to a healthy, spherical optic cup. Failure of the optic fissure to close gives rise to an ocular disorder known as coloboma. During this developmental process, *Foxg1* is expressed in the optic neuroepithelium, with highest levels of expression in the nasal optic stalk. *Foxg1*^−/−^ mutant mice have microphthalmic eyes with a large ventral coloboma. We found *Wnt8b* expression upregulated in the *Foxg1*^−/−^ optic stalk and hypothesized that, similar to what is observed in telencephalic development, Foxg1 directs development of the optic neuroepithelium through transcriptional suppression of *Wnt8b*. To test this, we generated *Foxg1*^−/−^*;Wnt8b*^−/−^ double mutants of either sex and found that the morphology of the optic cup and stalk and the closure of the optic fissure were substantially rescued in these embryos. This rescue correlates with restored Pax2 expression in the anterior tip of the optic fissure. In addition, although we do not find evidence implicating altered proliferation in the rescue, we observe a significant increase in apoptotic cell density in *Foxg1*^−/−^*;Wnt8b*^−/−^ double mutants compared with the *Foxg1*^−/−^ single mutant. Upregulation of Wnt/β-catenin target molecules in the optic cup and stalk may underlie the molecular and morphological defects in the *Foxg1*^−/−^ mutant. Our results show that proper optic fissure closure relies on *Wnt8b* suppression by Foxg1 in the nasal optic stalk to maintain balanced apoptosis and Pax2 expression in the nasal and temporal edges of the fissure.

**SIGNIFICANCE STATEMENT** Coloboma is an ocular disorder that may result in a loss of visual acuity and accounts for ∼10% of childhood blindness. It results from errors in the sealing of the optic fissure (OF), a transient structure at the bottom of the eye. Here, we investigate the colobomatous phenotype of the *Foxg1*^−/−^ mutant mouse. We identify upregulated expression of Wnt8b in the optic stalk of *Foxg1*^−/−^ mutants before OF closure initiates. *Foxg1*^−/−^*;Wnt8b*^−/−^ double mutants show a substantial rescue of the *Foxg1*^−/−^ coloboma phenotype, which correlates with a rescue in molecular and cellular defects of *Foxg1*^−/−^ mutants. Our results unravel a new role of Foxg1 in promoting OF closure providing additional knowledge about the molecules and cellular mechanisms underlying coloboma formation.

## Introduction

Vertebrate eye development is a multistep process that involves early specification of the eye field followed by bilateral evagination of the diencephalon, giving rise to the optic vesicle. The optic vesicle will then invaginate and form the optic stalk (OS) ventrally and the two-layered optic cup (OC), with an outer layer known as retinal pigment epithelium (RPE) and the inner (close to the lens) neural retina. As the OC grows, the apposed edges of its inferior part, known as the optic fissure (OF), come in close proximity and fuse together to give rise to a fully formed spherical eye structure (Chow and Lang, 2001; Lamb et al., 2007; Fuhrmann, 2010).

The cellular and molecular mechanisms that control OF closure are not fully understood. Errors underlying this process lead to an ocular disorder known as coloboma. Although environmental factors have been implicated in defective OF closure, it is well established that mutations in genes that are normally expressed in the optic vesicle give rise to coloboma (Gregory-Evans et al., 2004; Chang et al., 2006; Williamson and FitzPatrick, 2014).

The study of animal models with colobomatous phenotypes has allowed a better understanding regarding the cellular and molecular basis of the disorder. Among the cellular processes underlying OC formation and OF closure are cell proliferation and apoptotic cell death.

Among the molecules that are involved in coloboma formation is the transcription factor Pax2. Mutations in the *PAX2* gene in humans lead to the renal-coloboma syndrome, characterized by renal and ocular malformations, including optic nerve coloboma (Schimmenti et al., 1995; Schimmenti, 2011). Loss of Pax2 in mice leads to a coloboma phenotype due to inability of the edges of the OF to fuse (Torres et al., 1996). New molecular players are continuously added to the list of genes leading to coloboma in mice and humans (Gregory-Evans et al., 2004; Chang et al., 2006; Williamson and FitzPatrick, 2014), including proteins implicated in the HH (Wen et al., 2015), Fgf (Cai et al., 2013), Bmp (Huang et al., 2015), RA (Lupo et al., 2011), and Wnt (Liu and Nathans, 2008; Zhou et al., 2008; Lieven and Rüther, 2011; Liu et al., 2012, 2016; Alldredge and Fuhrmann, 2016) signaling pathways.

*Foxg1* is forkhead box transcription factor expressed from early stages of mouse embryonic development in the developing nervous system and is specifically found in the telencephalon, optic chiasm, and retina (Xuan et al., 1995; Huh et al., 1999; Pratt et al., 2004; Fotaki et al., 2006, 2013; Tian et al., 2008). Mice with no functional Foxg1 (*Foxg1*^−/−^ mutants) die at birth and show severe reduction in the size of telencephalic lobes and eyes (Xuan et al., 1995). In addition, *Foxg1*^−/−^ eyes display a large ventral coloboma (Huh et al., 1999). Foxg1's role in the developing eye has not been studied in detail. We have recently shown that, in the mouse, Foxg1 is essential for controlling the size of the ciliary margin in the nasal peripheral retina and for suppressing Wnt/β-catenin signaling in this region (Fotaki et al., 2013).

Here, we examine the molecular and cellular causes of the coloboma phenotype of the *Foxg1*^−/−^ mutant. We found that *Wnt8b* expression in the wild-type OS is upregulated in this mutant. We hypothesized that, similar to the telencephalon (Danesin et al., 2009), Foxg1 may normally suppress *Wnt8b* in the nasal OS for proper OC and/or OS formation to take place. We tested this by suppressing Wnt8b expression genetically in *Foxg1*^−/−^*;Wnt8b*^−/−^ double mutants. In accordance with our hypothesis, in the double mutant, we found substantial rescue of the OF closure defect we observed in the *Foxg1*^−/−^ single mutant. Our results reveal a novel mechanism of OF closure, which relies on Foxg1-mediated suppression of *Wnt8b* in the nasal OS to maintain balanced apoptosis and normal Pax2 expression in the nasal edges of the fissure.

## Materials and Methods

### 

#### 

##### Mice.

All experiments were done according to Home Office regulations (Edinburgh, United Kingdom).

*Foxg1*^+/−^ heterozygote males were bred to F1 (CBAxC57/B6) females to produce *Foxg1*^+/−^ heterozygous males and females as previously described (Fotaki et al., 2013). The *Wnt8b*^+/−^ mice have been previously described (Fotaki et al., 2010). *Foxg1*^+/−^*;Wnt8b*^+/−^ double heterozygotes were generated by intercrossing *Foxg1*^+/−^ and *Wnt8b*^+/−^ heterozygous mice.

##### Embryos.

To generate *Foxg1*^−/−^ single or *Foxg1*^−/−^*;Wnt8b*^−/−^ double-mutant embryos, timed matings were set up among *Foxg1*^+/−^ heterozygote or *Foxg1*^+/−^*;Wnt8b*^+/−^ double heterozygote male and female mice, respectively. The day the vaginal plug was detected in females was designated as E0.5.

No gross differences were detected in morphology or marker expression between wild-types (*Foxg1*^+/+^) and Foxg1^+/−^ heterozygotes (data not shown). However, unless otherwise stated, in most cases when comparing *Foxg1*^−/−^ homozygotes with controls, we used wild-type embryos.

*Foxg1*^−/−^*;Wnt8b*^−/−^ double-mutant embryos were compared with two experimental groups: (1) a control group, consisting of wild-type (*Foxg1*^+/+^*;Wnt8b*^+/+^), single heterozygote (*Foxg1*^+/−^*;Wnt8b*^+/+^ *or Foxg1*^+/+^*;Wnt8b*^+/−^), or double heterozygote (*Foxg1*^+/−^*;Wnt8b*^+/−^) embryos; or (2) a group where the *Foxg1* mutation was found in homozygosis (*Foxg1*^−/−^) and the *Wnt8b* allele was either wild-type (*Wnt8b*^+/+^) or heterozygous (*Wnt8b*^+/−^). This group was collectively named as the *Foxg1*^−/−^*;Wnt8b*^+/±^ mutant group.

##### Genotyping of mice and embryos.

*Foxg1*^+/−^ heterozygote mice express one copy of functional β-galactosidase under the control of the Foxg1 promoter and were distinguished from wild-types (*Foxg1*^+/+^) by PCR analysis based on detection of the lacZ allele (Xuan et al., 1995). For all embryos younger than E11.0, the *Foxg1* mutation was detected by PCR using primers specific for the *Foxg1*-wild-type allele (Foxg1-wt-F: AGG CTG ACG CAC TTG GAG; Foxg1-wt-R: CAG GGG TTG AGG GAG TAG GT), resulting in an 856 bp PCR product and for the *Foxg1*-null allele (Foxg1-mt-F: GCT GGA CAT GGG AGA TAG GA; Foxg1-mt-R: GAC AGT ATC GGC CTC AGG AA), resulting in a 650 bp PCR product. For embryos older than E11.0, *Foxg1*^−/−^ mutants were clearly distinguished by their phenotype as previously described (Fotaki et al., 2013).

The *Wnt8b* mutation was detected by PCR in both mouse and embryonic tissue as previously described (Fotaki et al., 2010).

##### Histology and cresyl violet staining.

Mouse embryos were collected on ice-cold PBS buffer and fixed in 4% PFA in 0.1 m phosphate buffer as previously described (Fotaki et al., 2006). Embryos were either embedded in a 1:1 mixture of OCT/sucrose (30%) for cryostat sectioning or in paraffin for microtome sectioning (Fotaki et al., 2013). Embryos used for cell counts were embedded in paraffin and cut at 7 μm horizontal sections. Some sections were stained with 0.2% of cresyl violet acetate (Sigma-Aldrich).

##### Riboprobe synthesis, *in situ* hybridization, immunohistochemistry, immunofluorescence, and X-gal staining.

Probes were labeled with digoxigenin according to the manufacturer's instructions (Roche). Riboprobes used for this study were for *Axin2* (Fotaki et al., 2011), *Bmp7* (Morcillo et al., 2006), *Celsr3* (Tissir et al., 2005), *Foxg1* (Fotaki et al., 2013), *Foxd1* (Herrera et al., 2004), *Fzd3* (Montcouquiol et al., 2006), *Vangl* (Montcouquiol et al., 2006), *Vax1* (Bertuzzi et al., 1999), *Wnt2b* (Fotaki et al., 2013), *Wnt3a*, *Wnt5a* (Liu et al., 2003; Ang et al., 2004), and *Wnt7b*, *Wnt8b*, as described by Fotaki et al. (2011).

Previously described protocols were used for *in situ* hybridization, immunohistochemistry, immunofluorescence, and X-gal staining (Fotaki et al., 2008, 2011, 2013). Pax2 DAB immunohistochemistry was performed on already X-gal-stained sections of *Foxg1*^+/−^ embryos (see Fig. 11*A*,*B*). DAB immunohistochemistry for β-gal was performed on sections that had already been reacted for *Wnt8b in situ* hybridization (see Fig. 3). Following incubation with the appropriate secondary biotinylated antibody, DAB immunohistochemistry was performed using the Vectastain ABC kit (Vector Laboratories), as previously described (Fotaki et al., 2006). *In situ* hybridization sections were in many cases counterstained with Nuclear Fast Red (Vector Laboratories). Immunofluorescence-reacted sections were counterstained with DAPI dilactate (2 μg/ml) (Sigma-Aldrich). All experiments were performed on at least 6 eyes from 3 different embryos for each experimental group (unless otherwise indicated).

All antibodies used for experiments are listed in Table 1. All Alexa-fluorescent antibodies were from Invitrogen/ThermoFisher Scientific.

**Table 1.** Antibodies used in this study

Primary antibody	Source	Secondary antibody	Reference
β-gal RRID:AB_221539	Rabbit; ThermoFisher Scientific (A11132)	Goat anti-rabbit biotinylated (Vector Laboratories)	Fotaki et al., 2011
β-tubulin III (Tuj1) RRID:AB_477590	Mouse; Sigma-Aldrich (T8660)	Donkey anti-mouse Alexa-568-IgG (H + L)	Fotaki et al., 2006
BrdU RRID:AB_10015219	Mouse, clone B44; BD Biosciences (347580)	Goat anti-mouse Alexa-488-IgG1	Martynoga et al., 2005
BrdU RRID:AB_305426	Rat; Abcam (ab6326)	Donkey anti-rat Alexa-488-IgG (H + L)	Fotaki et al., 2013
cleaved Caspase-3 RRID:AB_2070042	Rabbit; Cell Signaling Technology (9664)	Goat anti-rabbit biotinylated (Vector Laboratories)	Noh et al., 2016
Chx10 (Vsx2) RRID:AB_262173	Sheep; Millipore (AB9014)	Donkey anti-sheep Alexa-488	Klimova and Kozmik, 2014
Coup-TFI RRID:AB_1964211	Mouse; R&D Systems (PP-H8132-00)	Goat anti-mouse Alexa-488-IgG (H + L)	Tang et al., 2010
Coup-TFII RRID:AB_1964214	Mouse; R&D Systems (PP-H7147-00)	Goat anti-mouse Alexa-488-IgG (H + L)	Tang et al., 2010
Islet-1 RRID:AB_1157901	Mouse; DSHB (39.3F7)	Donkey anti-mouse Alexa-568-IgG (H + L)	Fotaki et al., 2006
Histone H3 (phospho S10) RRID:AB_304763	Rabbit; Abcam (ab5176)	Goat anti-rabbit Alexa-488-IgG (H + L)	Sargeant et al., 2008
c-Jun RRID:AB_2130165	Rabbit; Cell Signaling Technology (60A8)	Donkey anti-rabbit Alexa-488-IgG (H + L)	Cell Signaling Technology
Mitf RRID:AB_298801	Mouse; Abcam (ab12039)	Goat anti-mouse Alexa-488-IgG (H + L)	Abcam review
Mitf	Rabbit; Prof. Arnheiter's laboratory	Goat anti-rabbit Alexa-568-IgG (H + L)	Nguyen and Arnheiter, 2000
pMLC2 RRID:AB_374325	Rabbit; GeneTex (GTX22480)	Goat anti-rabbit Alexa-568-IgG (H + L)	Eiraku et al., 2011
Pax2 RRID:AB_2533990	Rabbit; ThermoFisher Scientific (71-6000)	Goat anti-rabbit Alexa-568-IgG (H + L)	Burns et al., 2008
Pax6 RRID:AB_2315070	Mouse; DSHB	Goat anti-mouse Alexa-488-IgG1	Fotaki et al., 2006
Pax6 RRID:AB_291612	Rabbit; Covance (PRB-278P)	Goat anti-rabbit Alexa-568-IgG (H + L)	Cai et al., 2010

##### Imaging.

DAB and *in situ* images were taken with a Leica DFC480 camera connected to a Leica DMNB epifluorescence microscope. Fluorescence images were taken with a Leica DM5500B automated upright microscope connected to a DFC360FX camera. Whole embryonic eyes were photographed with a Leica DFC420 camera connected to a Leica M165C stereomicroscope, all from Leica Microsystems. Confocal images were taken with an LSM 510 Axioskop (Leica Microsystems).

##### Labeling index (LI), mitotic index, apoptotic cell density, and Pax2 cell density counts.

To calculate the LI, we used *Foxg1*^+/+^ wild-type controls (*n* = 4 eyes from 3 different embryos) and *Foxg1*^−/−^ mutants (*n* = 5 eyes from 3 different embryos). Using confocal images, we counted the total number of cells counterstained with DAPI and the total number of cells immunostained with BrdU from 3 or 4 dorsal to ventral sections from the nasal and temporal components of the retina. Cell counts were performed manually on merged stacks of confocal images using the software IMARIS 8.0.0 (Bitplane, RRID:SCR_007370), which allows image rotation, facilitating counts in the *x-y-z*-axis. LI was calculated as the ratio of BrdU-positive to the total number of cells in the nasal and temporal retinae.

To calculate the pHH3 cell surface density (mitotic index), we used *Foxg1*^+/+^ wild-type controls (*n* = 4 eyes from 3 different embryos) and *Foxg1*^−/−^ mutants (*n* = 5 eyes from 3 different embryos). The total number of pHH3-positive cells was counted manually in the nasal and temporal retinae of each section from 3 or 4 dorsal to ventral sections. ImageJ (RRID:SCR_003070) was used to outline and calculate the perimeter of the apical surface of the nasal and temporal retinae, where counts were taken from and the volume of each section was calculated by multiplying the thickness of the section (7 μm) by the area.

To calculate the apoptotic cell density in our first experimental setup (Table 2), we used *Foxg1*^+/+^ wild-type controls (*n* = 5 eyes from 3 different embryos) and *Foxg1*^−/−^ mutants (*n* = 5 eyes from 3 different embryos); and in our second experimental setup (Table 3), we used control (*n* = 6 eyes from 4 different embryos), *Foxg1*^−/−^*;Wnt8b*^+/±^ (*n* = 4 eyes from 2 different embryos), and *Foxg1*^−/−^*;Wnt8b*^−/−^ double mutant (*n* = 4 eyes from 3 different embryos) embryos. We counted cleaved caspase-3-positive cells from 3 to 5 middle to ventral sections from each eye where OF was detectable and omitted dorsal sections, as cell death detection in these was absent from all our experimental groups. The area of the nasal and temporal retinae, where counts were taken from, was traced using ImageJ, and the volume of each section was calculated by multiplying the thickness of the section (7 μm) by the area.

**Table 2.** Apoptotic cell densities in *Foxg1*^+/+^ wild-type and *Foxg1*^−/−^ mutant nasal and temporal retinae*^a^*

Region	Cell density (×10^3^)	SE	97.5% CI
*Foxg1*^+/+^ nasal	1.16	0.12	0.89, 1.44
*Foxg1*^−/−^ nasal	0.42	0.05	0.31, 0.53
*Foxg1*^+/+^ temporal	0.98	0.42	0.03, 1.93
*Foxg1*^−/−^ temporal	1.19	0.3	0.52, 1.86

*^a^n* = 5 eyes from 3 different embryos for each group. Two cell densities are statistically different (*p* < 0.05) when the CIs do not intersect.

**Table 3.** Apoptotic cell densities in control, *Foxg1*^−/−^;*Wnt8b*^+/±^, and *Foxg1*^−/−^*;Wnt8b*^−/−^ nasal and temporal retinae*^a^*

Region	Cell density (× 10^3^)	SE	99.6% CI
Control nasal	1.55	0.30	(0.75, 2.34)
*Foxg1*^−/−^;*Wnt8b*^+/±^ nasal	0.27	0.11	(0.00, 0.54)
*Foxg1*^−/−^;*Wnt8b*^−/−^ nasal	0.63	0.02	(0.57, 0.69)
Control temporal	2.01	0.41	(0.92, 3.11)
*Foxg1*^−/−^;*Wnt8b*^+/±^ temporal	1.11	0.17	(0.66, 1.57)
*Foxg1*^−/−^;*Wnt8b*^−/−^ temporal	1.47	0.60	(0.00, 3.06)

*^a^n*^control^ = 6 eyes from 4 different embryos; *n*^*Foxg1*−/−;*Wnt8b*+±^ = 4 eyes from 2 different embryos; *n*^*Foxg1*−/−;*Wnt8b*−/−^ = 4 eyes from 3 different embryos. Two cell densities are statistically different (*p* < 0.05) when the CIs do not intersect.

To calculate the Pax2 cell density at the edges of the OF, we used control (*n* = 3 eyes from 3 different embryos); *Foxg1*^−/−^*;Wnt8b*^+/±^ mutant (*n* = 4 eyes from 3 different embryos) and *Foxg1*^−/−^*;Wnt8b*^−/−^ double mutant (*n* = 3 eyes from 3 different embryos) embryos. We counted Pax2-positive cells from 2 representative mid-sagittal sections within a square of 0.1 mm × 0.1 mm encompassing the edges of the fissure and the average values for the nasal and temporal retinae of each specimen were used for comparisons.

##### Corrected total cell fluorescence (CTCF) counts.

To quantitate Pax6 and Pax2 expression, we used controls (for E10.5: *n* = 5 eyes and for E11.5: *n* = 4 eyes from 3 different embryos); *Foxg1*^−/−^*;Wnt8b*^+/±^ mutants (for E10.5: *n* = 6 eyes and E11.5: *n* = 4 eyes from 3 different embryos) and *Foxg1*^−/−^*;Wnt8b*^−/−^ double mutants (for E10.5 and E11.5: *n* = 4 eyes from 3 different embryos) at E10.5 and E11.5. Using ImageJ, we outlined the nasal and temporal retinae; and for each section, we measured the area, the mean fluorescence, and the integrated fluorescent density, along with several adjacent background readings in 3–6 representative sections per specimen along the ventrodorsal axis. The most dorsal sections were not included in the study as their morphological differences in the mutants are more subtle than those observed in middle and ventral sections. For each section, we calculated the CTCF according to the following formula: CTCF = Integrated density − (Selected area × Mean fluorescence of background readings) (McCloy et al., 2014). The obtained values were divided by 1000.

##### Statistics.

To compare the LI, mitotic index, and apoptotic cell density between our different experimental groups, we assumed that this follows a normal distribution within the mouse population of the same genotype and that two values were different when the CIs did not intersect. The CIs were calculated using the mean values (LI, mitotic index, or apoptotic cell density), the SE, and the value of the normal distribution that corresponds to at least 95% confidence level (*p* ≤ 0.05), after applying the Bonferroni correction where appropriate. For our calculations, we used a sampling strategy. We used a two-stage sampling strategy (Shiver and Borders, 1996), in which the primary units are the mouse eyes and the secondary units are the eye sections. This sampling strategy, a widely used methodology in experimental fields such as Forestry, is applied, for example, to calculate total number of trees and/or ratios among specific species (Shiver and Borders, 1996).

Data analysis was performed using R software (version 3.1.2, RRID:SCR_001905) and the package survey for analysis of complex surveys (http://r-survey.r-forge.r-project.org/survey/). The package survey produces an estimate of the mean ratio (LI = Brdu-positive cells/total number of cells; cell density = cell count/volume) and its SE.

To compare the Pax6 and Pax2 CTCF values, we performed ANOVA followed by Bonferroni correction, using the averaged CTCF values along the ventrodorsal axis for the nasal and temporal retina of each specimen analyzed. A similar procedure was followed to calculate the Pax2-positive cell density in the nasal and temporal retinae. The degrees of freedom (df), the *F* value, and the *p* value were calculated using the software SPSS (IBM, SPSS Statistics 21, RRID:SCR_002865).

##### RNA extraction, cDNA synthesis, and PCR arrays.

The OCs of E11.0 wild-type and *Foxg1*^−/−^ age-matched embryos were dissected out using fine-tip (0.125 mm) tungsten-dissecting probes (WPI) and snap frozen on dry ice. RNA was generated pooling together 6 eyes, dissected out from 3 to 5 different wild-type or *Foxg1*^−/−^ mutant embryos, and was extracted using the RNeasy micro kit (QIAGEN) following the manufacturer's instructions; 150 ng of RNA from three independent samples of *Foxg1*^+/+^ wild-type and *Foxg1*^−/−^ mutant OCs was used to synthesize cDNA, using the RT2 First Strand Kit (QIAGEN) according to the manufacturer's instructions. The cDNA was mixed with RT2 SYBR Green ROX qPCR mastermix (QIAGEN) according to the manufacturer's instructions, and the mixture was loaded on RT2 Profile PCR array plates for the mouse Wnt signaling pathway [PAMM-043Z] (QIAGEN). The plates were run on a StepOnePlus Real-Time PCR machine (Applied Biosystems/ThermoFisher Scientific). Results obtained from three plates for each group were further processed through the QIAGEN GeneGlobe Data Analysis Centre (http://www.qiagen.com/gb/shop/genes-and-pathways/data-analysis-center-overview-page/).

## Results

### Expression of *Foxg1* in the developing OC and OS at E10.5

**Figure 1.**
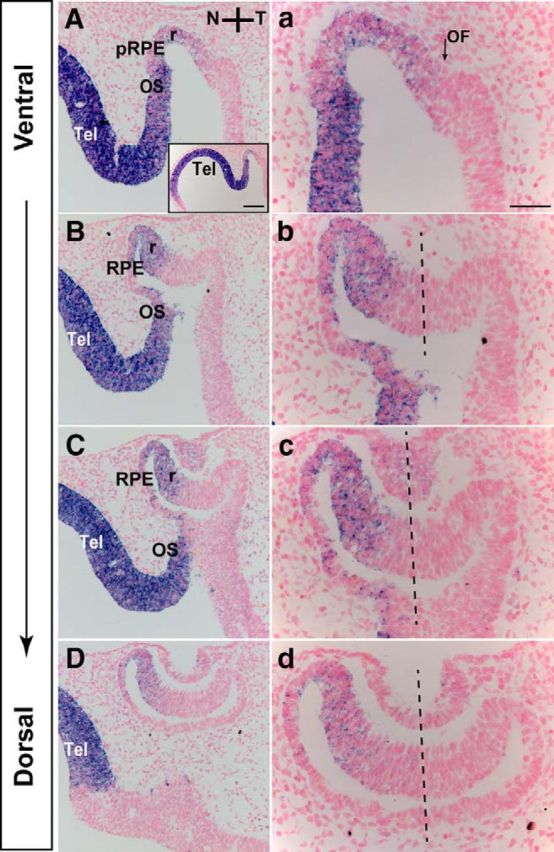
*Foxg1* mRNA expression in the developing optic neuroepithelium. In E10.5 horizontal sections along the ventral-to-dorsal axis, *Foxg1* is expressed in the nasal retina (r), RPE, and OS (***A–D***, ***a–d***). Strong *Foxg1* expression is detected in the telencephalon (Tel). ***a***, Arrow indicates the forming OF. N-T, Nasal-temporal axis (***b–d***, dashed lines). pRPE, Presumptive RPE. Scale bars: ***A–D***, 100 μm; ***a–d***, 50 μm; ***A***, Inset, 200 μm.

*Foxg1* expression in the developing mouse retina has been described in detail at E12.5 and E14.5, after completion of OF closure (Huh et al., 1999; Pratt et al., 2004; Tian et al., 2008; Fotaki et al., 2013). To gain understanding about *Foxg1* expression in the developing OC before the OF seals, we performed *in situ* hybridization at E10.5 on horizontal sections throughout the ventral-to-dorsal axis (Fig. 1). *Foxg1* expression was detected in the nasal retina and RPE (OC) and in the OS in agreement with previous observations (Hatini et al., 1994). *Foxg1* expression was higher in ventral and middle sections compared with dorsal sections (compare Fig. 1*a–c* with Fig. 1*d*). In ventral sections, *Foxg1* expression appeared more extended toward the midline of the nasotemporal axis than in middle and dorsal sections and reached the nasal edge of the OF (Fig. 1*a*, arrow). *Foxg1* expression in the OC was lower than that in the OS and telencephalon throughout the ventral-to-dorsal axis (i.e., compare intensity of telencephalic and OS to OC signal in Fig. 1*A–C*). Our results highlight high expression of Foxg1 in the nasal OS and ventral OC, both of which are involved in OF formation.

### Increased *Wnt8b* expression in the *Foxg1*^−/−^ OS at E10.5

In the mouse telencephalon, Foxg1 suppresses expression of Wnt molecules (*Wnt2b*, *Wnt3a*, *Wnt5a*, *Wnt7b*, and *Wnt8b*), normally confined to the dorsomedial telencephalon and/or cortical hem (Muzio and Mallamaci, 2005; Hanashima et al., 2007; and data not shown). We hypothesized that the same Wnt molecules are also upregulated in the developing optic vesicle leading to defects in OF closure. Using *in situ* hybridization, we examined expression of these Wnts in wild-type E12.0-E12.5 horizontal sections to determine expression in the OC and OS.

**Figure 2.**
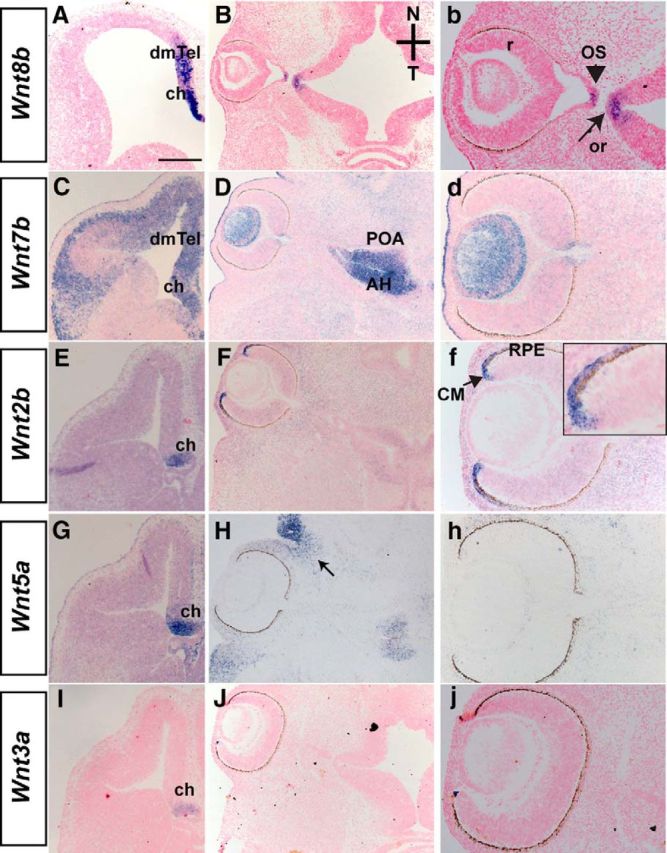
Expression of Wnt molecules in the developing telencephalon and optic neuroepithelium. E12.5 wild-type horizontal sections depicting expression of *Wnt8b* (***A***), *Wnt7b* (***C***), *Wnt2b* (***E***), *Wnt5a* (***G***), and *Wnt3a* (***I***) in the dorsomedial telencephalon (dmTel) and cortical hem (ch). *Wnt8b* is detected in the OS (***B***, ***b***, arrowhead) and the hypothalamic optic recess (or) (***b***, arrow). *Wnt7b* is expressed in the lens (***D***, ***d***). *Wnt7b* is also expressed strongly in the diencephalic preoptic area (POA) and anterior hypothalamus (AH) (***D***). *Wnt2b* is expressed in the ciliary margin (CM) and peripheral RPE (***F***, ***f***). *Wnt5a* is detected in the eyelid epithelium (***H***, arrow) but, similar to *Wnt3a*, it is not detected within the optic neuroepithelium (***H***, ***h***, ***J***, ***j***). Scale bars: ***A***, ***C***, ***E***, ***G***, ***I***, 400 μm; ***B***, ***D***, ***F***, ***H***, ***J***, 200 μm; ***b***, ***d***, ***f***, ***h***, ***j***, 100 μm.

All five Wnt molecules examined were expressed in the dorsomedial telencephalon and/or cortical hem (Fig. 2*A*,*C*,*E*,*G*,*I*), as previously described (Richardson et al., 1999; Muzio and Mallamaci, 2005; Fotaki et al., 2010; and data not shown). In the optic neuroepithelium, *Wnt8b* was detected in a small domain in the OS (Fig. 2*B*,*b*, arrowhead) (Roy et al., 2013). *Wnt7b*-positive cells were observed in the lens (Fig. 2*D*,*d*) (Liu et al., 2003; Ang et al., 2004), whereas *Wnt2b* expression was confined in the peripheral RPE and ciliary margin (Fig. 2*F*,*f*) (Liu et al., 2003; Fotaki et al., 2013). *Wnt5a* was detected in the eyelid epithelium (Fig. 2*H*,*h*), as previously reported (Liu et al., 2003; Kumar and Duester, 2010), whereas *Wnt3a* was not detected in any part of the developing OC or stalk or surrounding periocular mesenchyme (Fig. 2*J*,*j*) (Liu et al., 2003).

**Figure 3.**
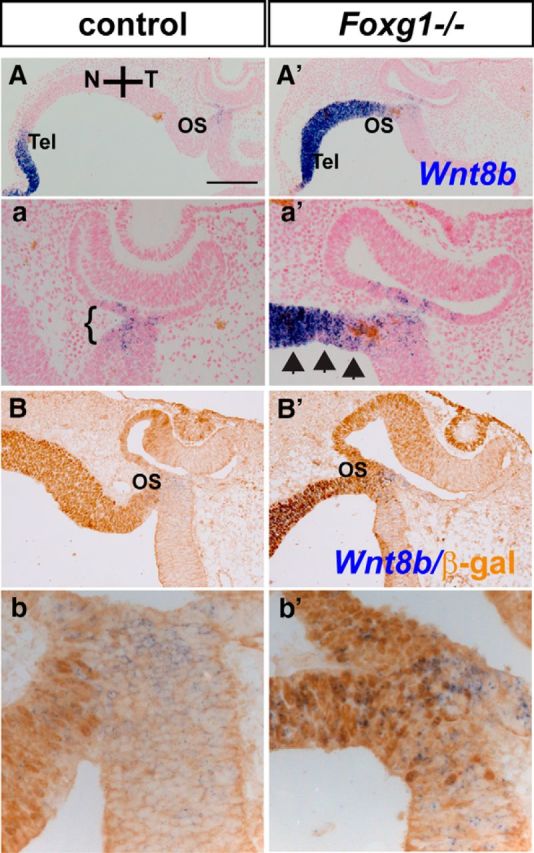
Upregulation of *Wnt8b* expression in the nasal OS in the *Foxg1*^−/−^ mutant at E10.5. *Wnt8b* is expressed in the dorsomedial telencephalon (Tel) (***A***) and in a small domain in the OS of controls (***a***, ***B***, ***b***, bracketed area) and is upregulated in the *Foxg1*^−/−^ mutant telencephalon (***A′***) and in the nasal OS (***a′***, ***B′***, ***b′***, arrows) where Foxg1 would normally be expressed (***B***, ***b***, ***B′***, ***b′***, β-gal staining). Scale bars: ***A***, ***A′***, 200 μm; ***a***, ***a′***, ***B***, ***B′***, 100 μm; ***b***, ***b′***, 50 μm.

We then studied expression of *Wnt2b*, *Wnt7b*, and *Wnt8b*, which were found expressed in the optic neuroepithelium, in E10.5 horizontal sections of control and *Foxg1*^−/−^ mutants by means of *in situ* hybridization. No differences were observed in *Wnt2b* and *Wnt7b* expression in the E10.5 OC between wild-types and *Foxg1*^−/−^ mutants (data not shown). Similar to E12.5, at E10.5 *Wnt8b* expression in the wild-type developing optic vesicle was restricted to a small domain in the OS (Fig. 3*A*,*a*). However in the *Foxg1*^−/−^ mutant, *Wnt8b* expression at and near the OS region was found expanded (Fig. 3*A′*,*a′*, arrowheads). In controls, *Wnt8b* expression was found in both the nasal and temporal OS; and nasally it was expressed in Foxg1-positive cells (reflected by positive β-gal staining, expressed under the control of the Foxg1 promoter; Fig. 3*B*,*b*). In *Foxg1*^−/−^ mutants, *Wnt8b* expansion was observed in the nasal OS, where Foxg1 is normally expressed (Fig. 3*B′*,*b′*).

Our results indicate that loss of Foxg1 leads to an upregulation of *Wnt8b* in the developing nasal OS, in a domain where Foxg1 is normally expressed, suggesting that Foxg1 may normally suppress Wnt8b function in this region.

### Significant rescue of the coloboma phenotype of the *Foxg1*^−/−^ mutant in a *Wnt8b*-null genetic background

Based on expansion of *Wnt8b* expression in the *Foxg1*^−/−^ OS at E10.5 (Fig. 3) and the previously described repressor activity of foxg1 on wnt8b in the zebrafish telencephalon (Danesin et al., 2009), we hypothesized that upregulated Wnt8b expression in the OS causes coloboma in the *Foxg1*^−/−^ mutant. To test our hypothesis, we crossed male and female mice, double heterozygous for the *Foxg1* (*Foxg1*^+/−^) (Xuan et al., 1995) and *Wnt8b* (*Wnt8b*^+/−^) (Fotaki et al., 2010) alleles, to generate double homozygous embryos *Foxg1*^−/−^*;Wnt8b*^−/−^.

**Figure 4.**
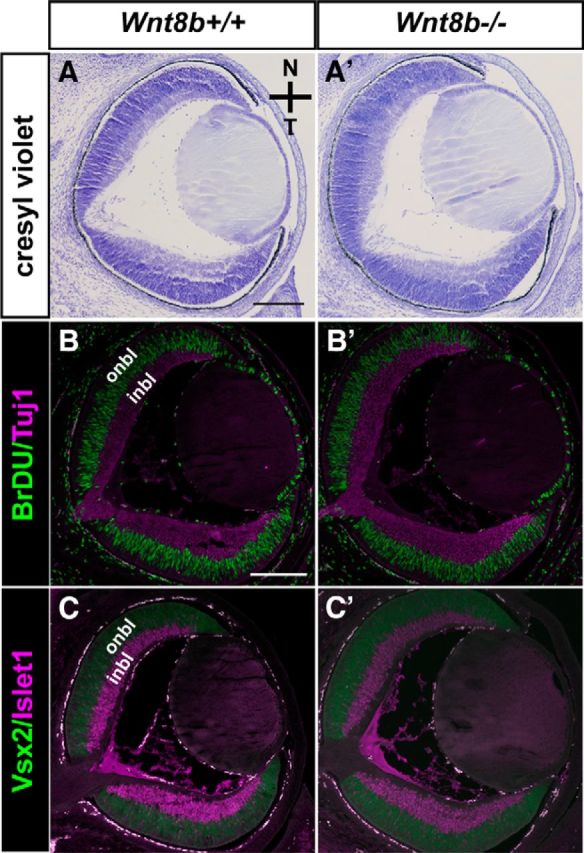
The *Wnt8b*^−/−^ null mutant shows normal OC morphology and marker expression. Horizontal sections of E15.5 wild-types (*Wnt8b*^+/+^) (***A–C***) and *Wnt8b*^−/−^ mutants (***A′–C′***) do not reveal any gross differences among genotypes. Cresyl violet wild-type (***A***) and *Wnt8b*^−/−^ mutant (***A′***) sections reveal normal OC morphology. Double immunofluorescence for BrdU and Tuj1 (***B***, ***B′***) and Vsx2 and Islet1 (***C***, ***C′***) shows that the proliferating, outer neuroblastic layer (onbl) (BrdU- and Vsx2-positive cells) and differentiating, inner neuroblastic layer (inbl) (Tuj1- and Islet1-positive cells) retinal layers are similar in wild-types (***B***, ***C***) and *Wnt8b*^−/−^ mutants (***B′***, ***C′***). Scale bars: ***A***, ***A′***, 200 μm; ***B***, ***B′***, ***C***, ***C′***, 200 μm.

We first assessed the phenotype of *Wnt8b*^−/−^ mutant OCs at E15.5. Cresyl violet staining showed similar OC morphology between wild-type and *Wnt8b*^−/−^ mutants and complete closure of the OF (Fig. 4*A*,*A′*). In addition, double immunofluorescence for markers of the proliferating (BrDU and Vsx2) and differentiating (Tuj1 and Islet1) retinal layers did not reveal any gross differences between wild-types and *Wnt8b*^−/−^ mutants (Fig. 4*B*,*B′*,*C*,*C′*).

**Figure 5.**
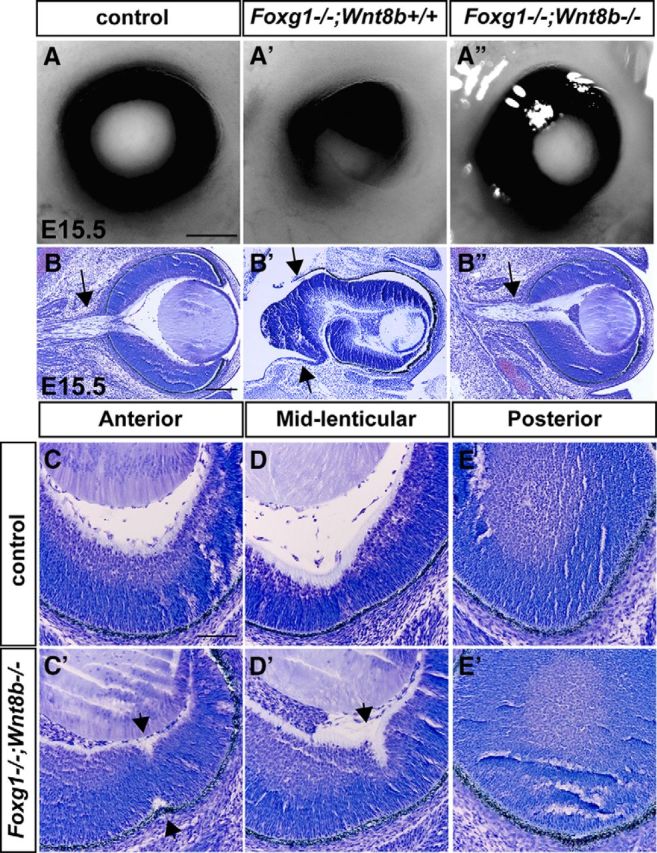
Rescue of the coloboma phenotype of the *Foxg1*^−/−^ mutant in a *Foxg1*^−/−^*;Wnt8b*^−/−^ null background. OC images of control embryos showing normal OF closure (***A***), *Foxg1*^−/−^*;Wnt8b*^+/+^ single mutants with a large ventral coloboma (***A′***), and *Foxg1*^−/−^*;Wnt8b*^−/−^ double mutants displaying rescue of the coloboma phenotype (***A″***). Cresyl violet-stained sections of control (***B***), *Foxg1*^−/−^*;Wnt8b*^+/+^ single (***B′***), and *Foxg1*^−/−^*;Wnt8b*^−/−^ double mutants (***B″***) revealed that the double-mutant OC and optic nerve (***B″***, arrow) resemble that of the control (***B***, arrow), rather than that of the single mutant (***B′***). In the single mutant, the OS does not form normally as indicated by ***B′*** (two arrows), resulting in abnormal formation of the optic nerve. High-power images of coronal sections of control (***C–E***) and *Foxg1*^−/−^*;Wnt8b*^−/−^ double mutants (***C′–E′***) in anterior (***C***, ***C′***), mid-lenticular (***D***, ***D′***), and posterior (***E***, ***E′***) levels show failure of the OF to seal completely in the double mutant anterior and mid-lenticular levels (***C′*** and ***D′***, respectively, arrowheads). Scale bars: ***A***, ***A′***, ***A″***, 20 μm; ***B***, ***B′***, ***B″***, 200 μm; ***C–E***, ***C′-E′***, 100 μm.

We then examined the eye phenotype of control, *Foxg1*^−/−^*;Wnt8b*^+/+^ or *Foxg1*^−/−^*;Wnt8b*^+/−^ (collectively designated as *Foxg1*^−/−^*;Wnt8b*^+/±^ mutants) and *Foxg1*^−/−^*;Wnt8b*^−/−^ double mutants at E15.5, when OF closure has normally been completed. Control eyes showed normal OC morphology and complete OF closure (Fig. 5*A*). *Foxg1*^−/−^*;Wnt8b*^+/±^ mutants displayed microphthalmia and ventral coloboma (Fig. 5*A′*), as previously described (Huh et al., 1999). As predicted by our hypothesis, *Foxg1*^−/−^*;Wnt8b*^−/−^ double mutants displayed a spherical-shaped OC and rescue of the coloboma phenotype (Fig. 5*A″*).

Gross analysis of OC morphology did not reveal a large ventral gap in any of the *Foxg1*^−/−^*;Wnt8b*^−/−^ double-mutant eyes examined (*n* = 12). Cresyl violet staining showed a spherical OC and optic nerve in control embryos (Fig. 5*B*), an abnormal OC with an elongated retina with foldings and with no clearly identifiable optic nerve in *Foxg1*^−/−^*;Wnt8b*^+/+^ mutants (100% of all eyes examined; *n* = 22) (Fig. 5*B′*) (Pratt et al., 2004) and an OC and optic nerve resembling that of controls in *Foxg1*^−/−^*;Wnt8b*^−/−^ double mutants (Fig. 5*B″*). Sequential coronal sections of *Foxg1*^−/−^*;Wnt8b*^−/−^ double-mutant eyes (*n* = 6) revealed that 50% of the OCs had complete OF closure anteriorly and at the mid-lenticular level and 100% at the posterior pole (Fig. 5*C′–E′*). At anterior and mid-lenticular levels, 50% of the OCs showed unfused tips in the form of small gaps or indentations (Fig. 5*C′*,*D′*), which were never observed in controls (*n* = 18 eyes) (Fig. 5*C*,*D*). The indentations anteriorly included both the interior and exterior aspects of the *Foxg1*^−/−^*;Wnt8b*^−/−^ neural retina (Fig. 5*C′*, arrowheads), whereas at mid-lenticular level it either included both (data not shown) or just the interior aspect (Fig. 5*D′*, arrowhead). The edges of the OF of the *Foxg1*^−/−^*;Wnt8b*^−/−^ OC at the posterior pole were fused and resembled those of controls (Fig. 5*E*,*E*′).

Our results collectively show a spectacular rescue of the OC and OS/nerve morphology and of the OF closure defect observed in the *Foxg1*^−/−^ mutant in a genetic background lacking *Wnt8b* expression and strongly suggest that *Wnt8b* expression normally needs to be suppressed by Foxg1 for normal OC and OS development to take place.

### Nasotemporal defects are not rescued in the *Foxg1*^−/−^*;Wnt8b*^−/−^ double-mutant retina

**Figure 6.**
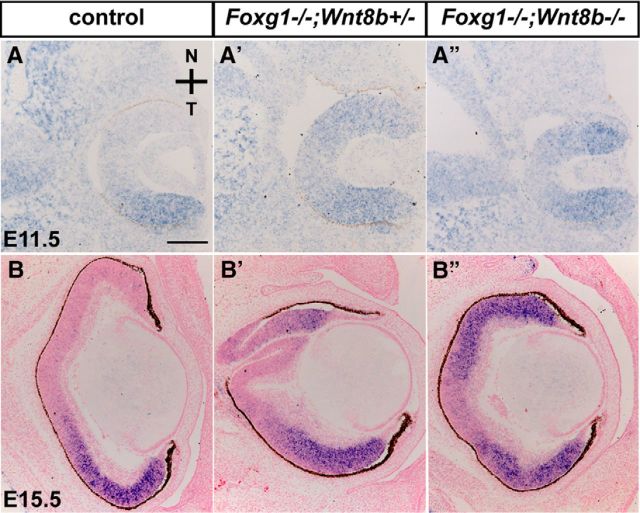
Upregulation of *Foxd1* expression in the *Foxg1*^−/−^ and in the *Foxg1*^−/−^*;Wnt8b*^−/−^ double mutant nasal retina. *Foxd1* mRNA expression in horizontal E11.5 (***A–A″***) and E15.5 (***B–B″***) sections is detected in the temporal retina of controls (***A***, ***B***) but is found upregulated throughout the temporal and nasal retinal domains in *Foxg1*^−/−^*;Wnt8b*^+/±^ (***A′***, ***B′***) and *Foxg1*^−/−^*;Wnt8b*^−/−^ double mutants (***A″***, ***B″***). Scale bars: ***A***, ***A′***, ***A″***, 100 μm; ***B***, ***B′***, ***B″***, 200 μm.

Foxg1 is crucial for specification of the nasal retina, and its loss leads to an abnormal expansion of the temporal expression of Foxd1 nasally (Huh et al., 1999; Tian et al., 2008). We hypothesized that the rescue of the double-mutant *Foxg1*^−/−^*;Wnt8b*^−/−^ OC morphology (Fig. 5*B″*), may be the result of a rescue in nasotemporal retinal patterning. *In situ* hybridization for *Foxd1* expression at E11.5 and E15.5 revealed restricted expression in the temporal retina in controls (Fig. 6*A*,*B*) and expanded expression in the nasal domain of *Foxg1*^−/−^*;Wnt8b*^+/±^ retinae (Fig. 6*A′*,*B′*). In the *Foxg1*^−/−^*;Wnt8b*^−/−^ double mutant, we observed a similar expansion of *Foxd1* to that observed in the *Foxg1*^−/−^*;Wnt8b*^+/±^ retinae at E11.5 and E15.5 (Fig. 6*A″*,*B″*). Results were similar at E10.5 and consistent for all specimens analyzed (8 eyes from 4 different embryos for each experimental group, across all ages), indicating that rescued morphology of the OC in *Foxg1*^−/−^*;Wnt8b*^−/−^ double mutants occurs despite the retention of nasotemporal patterning defects.

### The *Foxg1*^−/−^*;Wnt8b*^−/−^ double-mutant OC morphology at E10.5 resembles that of the *Foxg1*^−/−^ single mutant

**Figure 7.**
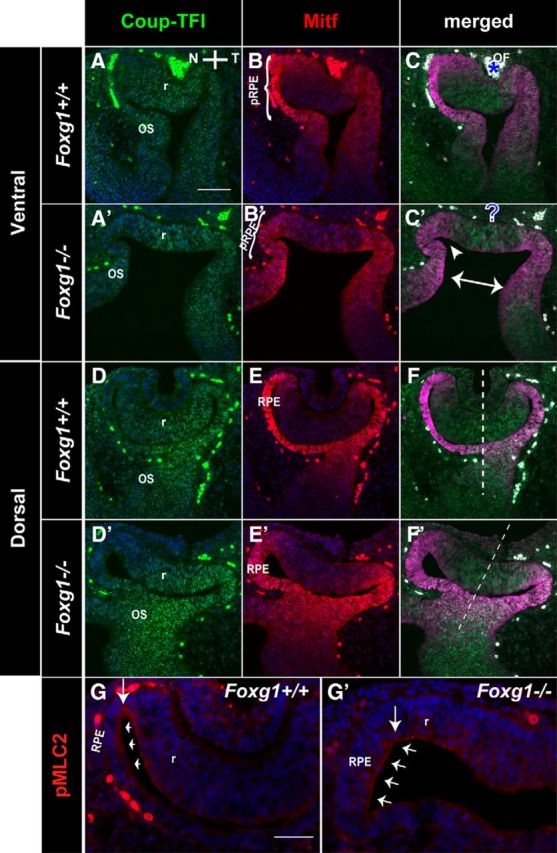
The shape of the nasal OC is compromised in *Foxg1*^−/−^ mutants. Double immunofluorescence for Coup-TFI and Mitf in wild-type (*Foxg1*^+/+^) ventral (***A–C***) and dorsal (***D–F***) and *Foxg1*^−/−^ ventral (***A′–C′***) and dorsal (***D′–F′***) E10.5 horizontal sections. Coup-TFI is found in the ventral (***A***, ***A′***) and dorsal (***D***, ***D′***) retina (r) and in the OS in both wild-types (***A***, ***D***) and *Foxg1*^−/−^ mutants (***A′***, ***D′***). Mitf is found in the RPE in ventral and dorsal sections in both wild-types (***B***, ***E***) and *Foxg1*^−/−^ mutants (***B′***, ***E′)***. ***B***, ***B′***, Brackets indicate the presumptive RPE (pRPE) in wild-types and *Foxg1*^−/−^ mutants, respectively. ***C***, *OF in wild-types, which is not clearly visible in *Foxg1*^−/−^ mutants (***C′***, ?). ***C′***, Double arrow indicates the abnormally enlarged distance between nasal and temporal OS in the *Foxg1*^−/−^ mutant. Arrowhead indicates the lack of invagination of the nasal OC. ***F***, ***F′***, Dashed lines indicate the nasotemporal axis. pMLC2 expression in wild-types (***G***) and *Foxg1*^−/−^ mutants (***G′***) is detected along the RPE (***G***, ***G′***, small arrows) and in the hinge region (***G***, ***G′***, vertical arrow). Scale bars: ***A–F***, ***A′–F′***, 100 μm; ***G***, ***G′***, 50 μm.

To understand when in development we first start to observe morphological differences between controls, *Foxg1*^−/−^*;Wnt8b*^+/±^ mutants and *Foxg1*^−/−^*;Wnt8b*^−/−^ double mutants, we examined the morphology and marker expression of the optic neuroepithelium in these experimental groups at E10.5. Using as markers Coup-TFI, which labels the retina and the OS (Tang et al., 2010), and Mitf, which labels the RPE (Nguyen and Arnheiter, 2000), we first observed major morphological differences between wild-types and *Foxg1*^−/−^ single mutants along the nasotemporal axis (Fig. 7). Coup-TFI expression was detected throughout the ventral OC (Fig. 7*A*,*A′*), whereas in dorsal sections it showed a ^high^temporal-^low^nasal gradient (Fig. 7*D*,*D*) (Tang et al., 2010) in wild-types and *Foxg1*^−/−^ mutants. Coup-TFI expression revealed that in ventral sections the *Foxg1*^−/−^ OC displayed an abnormal flattened shape (Fig. 7*A′*) compared with the wild-type U-shaped OC (Fig. 7*A*). In addition, the forming OF in wild-types (Fig. 7*C*, asterisk) was not detectable in the Foxg1^−/−^ mutant (Fig. 7*C′*, ?). Coup-TFI expression in the OS, although similar between wild-types and *Foxg1*^−/−^ mutants, revealed an abnormally enlarged distance between nasal and temporal OS in the *Foxg1*^−/−^ mutant (Fig. 7*C′*, double arrow). Regarding Mitf expression, this was limited to the RPE in wild-types and *Foxg1*^−/−^ mutants (Fig. 7*B*,*B′*,*E*,*E′*) but revealed a thickened nasal RPE in the *Foxg1*^−/−^ mutant in both ventral (Fig. 7*B′*) and dorsal (Fig. 7*E′*) sections.

We then examined our three experimental groups described above using well-established markers for the retina, RPE, and OS [Pax6: in the retina with ^high^peripheral-to-^low^central gradient and a ^low^ventral-to-^high^dorsal gradient and in the RPE (Walther and Gruss, 1991; Bäumer et al., 2003)]; [Coup-TFII: in the RPE and OS (Tang et al., 2010; Eiraku et al., 2011)]; [Pax2: in the OS and central retina with a ^high^ventral-to-^low^dorsal gradient (Nornes et al., 1990; Püschel et al., 1992; Bäumer et al., 2003)] to determine differences in optic neuroepithelium morphology and marker expression. Results described below were consistent for all specimens from the same experimental group (6 eyes from 3 different embryos for each experimental group).

**Figure 8.**
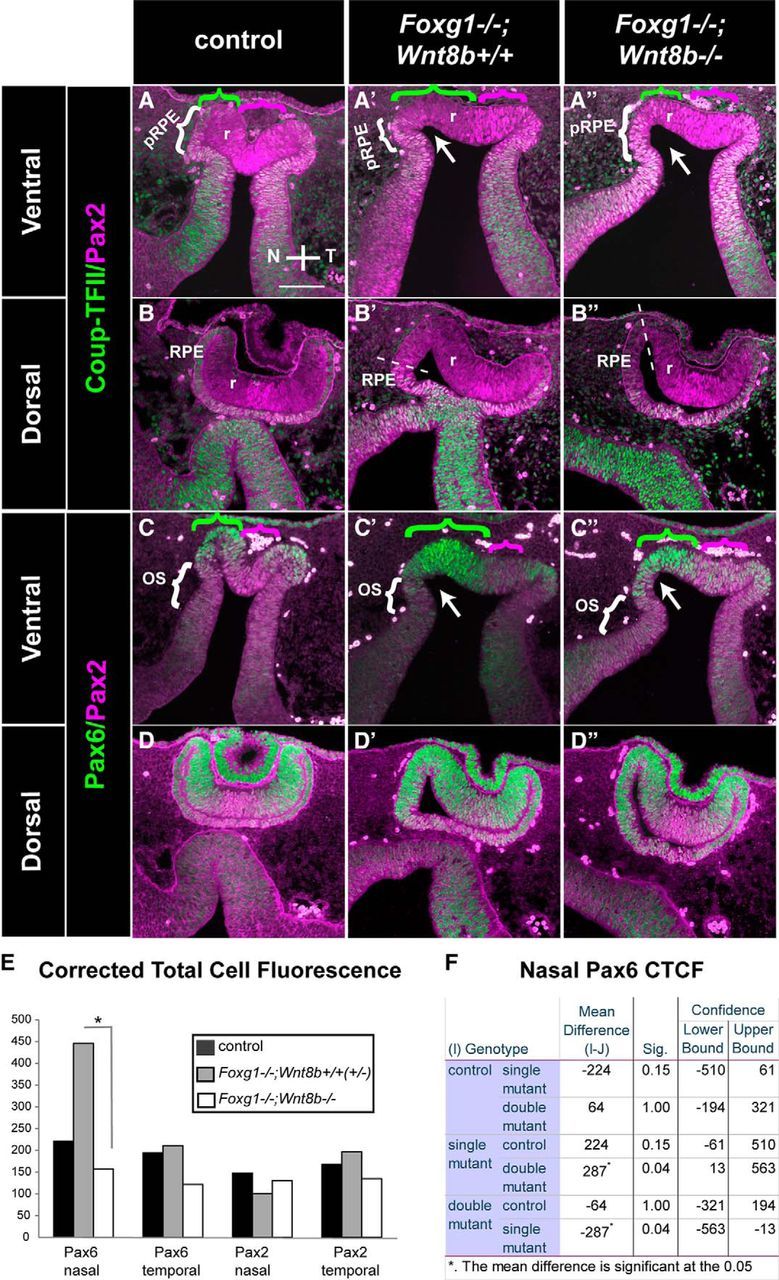
Marker analysis of the optic neuroepithelium in control, *Foxg1*^−/−^*;Wnt8b*^+/+^, and *Foxg1*^−/−^*;Wnt8b*^−/−^ E10.5 horizontal sections. Coup-TFII (green) and Pax2 (magenta) immunofluorescence in control (***A***, ***B***), *Foxg1*^−/−^*;Wnt8b*^+/+^ (***A′***, ***B′***), and *Foxg1*^−/−^*;Wnt8b*^−/−^ (***A″***, ***B″***) sections. Coup-TFII is found in the OS and the presumptive RPE (pRPE), indicated in ***A***, ***A′***, ***A″*** (brackets) in ventral sections and the RPE in dorsal sections (***B***, ***B′***, ***B″***) in controls (***A***, ***B***), single (***A′***, ***B′***) and double mutants (***A″***, ***B″***). Pax2 is found in the OS (***A***, ***C***) and in the region of the retina (r) that surrounds the forming OF in control ventral sections (***A***, ***C***, magenta bracketed areas). In *Foxg1*^−/−^*;Wnt8b*^+/+^ single-mutant ventral sections, Pax2 is found in the OS (***A′***, ***C′***), but expression in the retina is shifted toward the temporal domain, as indicated in ***A′***, ***C′*** (magenta bracket). In the *Foxg1*^−/−^*;Wnt8b*^−/−^ double mutant, Pax2 is expressed in the OS (***A″***, ***C″***) and throughout the retina in ventral sections (***A″***, ***C″***, magenta bracket). In dorsal sections, Pax2 expression is found in the region that will give rise to the optic disc (OD) (***B***, ***D***, ***B′***, ***D′***, ***B″***, ***D″***) in all three genotypes. Dorsal Pax2 expression in the *Foxg1*^−/−^*;Wnt8b*^+/+^ mutant is shifted to the temporal retina, as with ventral sections (***B′***, ***D′***). Pax6 (green) and Pax2 (magenta) immunofluorescence in control (***C***, ***D***), *Foxg1*^−/−^*;Wnt8b*^+/+^ (***C′***, ***D′***), and *Foxg1*^−/−^*;Wnt8b*^−/−^ (***C″***, ***D″***) sections. Pax6 expression is found in the retina and RPE throughout the ventrodorsal axis. In *Foxg1*^−/−^*;Wnt8b*^+/+^ mutants, the size of the ventronasal Pax6+ domain is clearly enlarged (***C′***, green bracket) compared with that of controls (***C***) and *Foxg1*^−/−^*;Wnt8b*^−/−^ double mutants (***C″***, green bracket). ***A***, ***A′***, ***A″***, Green brackets indicate nasal Pax2-negative expression, which corresponds to Pax6-positive expression in ***C***, ***C′***, ***C″***, whereas the magenta brackets indicate the Pax2 expression domain. ***A′***, ***C′***, ***A″***, ***C″***, Arrows indicate the lack of a clear constriction where the OC invagination forms nasally in single and double mutants, respectively. ***E***, Graph represents the difference in the mean values of the CTCF for Pax6 and Pax2 in the nasal and temporal retinal domains along the ventrodorsal axis. *p = 0.04. An ANOVA was performed to define the statistical significance of the difference of the results (***F***). *n*^control^ = 5 eyes from 3 different embryos; *n*^*Foxg1*−/−*;Wnt8b*+/+^ = 6 eyes from 3 different embryos; *n*^*Foxg1*−/−*;Wnt8b*−/−^ = 4 eyes from 3 different embryos. Scale bars, 100 μm.

In terms of morphology, the ventral OC of the *Foxg1*^−/−^*;Wnt8b*^−/−^ double mutant was similar to that of the *Foxg1*^−/−^*;Wnt8b*^+/+^ mutant, showing a flattened appearance and lack of the U-shape formation in controls due to lack of nasal invagination of the OC (Fig. 8*A″*,*C″*, arrows, *A′*,*C′*). However, in dorsal sections, *Foxg1*^−/−^*;Wnt8b*^−/−^ double-mutant OCs resembled the control OC shape (Fig. 8*B*,*B″*,*D*,*D″*). To determine whether the changes in *Foxg1*^−/−^ OC morphology may be attributed to defects in the formation of the “hinge” region at the nasal RPE-retinal transition, we examined expression of the phosphorylated myosin light chain 2 (pMLC2), which has been implicated in RPE stiffness and in shaping the OC (Eiraku et al., 2011; Carpenter et al., 2015). However, we did not observe any gross differences in pMLC2 expression between E10.5 wild-type and *Foxg1*^−/−^ mutant OCs at the hinge region that may account for the changes observed in the mutant nasal OC shape (Fig. 7*G*,*G′*).

In terms of molecular profile, our marker analysis revealed that, in the *Foxg1*^−/−^*;Wnt8b*^+/+^ single mutant, the Pax2-positive domain seemed reduced in the nasal retina in ventral sections compared with that of controls (compare areas indicated by magenta brackets in Fig. 8*A*,*C* and Fig. 8*A′*,*C′*), whereas Pax6 expression seemed expanded nasally in both ventral and dorsal sections compared with that of controls (compare areas indicated by green brackets in Fig. 8*C*,*C′* and Fig. 8*D–D′*). However, in *Foxg1*^−/−^*;Wnt8b*^−/−^ double mutants, Pax2 expression ventrally expanded throughout the nasal and temporal retinal domain (Fig. 8*A″*, magenta bracketed area), similar to that of controls, whereas Pax6 staining was restricted to the peripheral retina both nasally and temporally and resembled more that of control staining (Fig. 8*C″*, green bracketed area, *D″*).

To quantitate the above observations, we measured the CTCF for the Pax6 and Pax2 cells in the nasal and temporal retinae (for details, see Materials and Methods). The Pax6 CTCF was found increased in the *Foxg1*^−/−^*;Wnt8b*^+/+^ single mutant compared with that of controls and *Foxg1*^−/−^*;Wnt8b*^−/−^ double mutants (ANOVA; df, 2; *F* = 4.433, *p* = 0.036), and the difference reached statistical significance between single and double mutants (Fig. 8*E*,*F*). In the case of Pax2, although the CTCF was found reduced in the *Foxg1*^−/−^*;Wnt8b*^+/+^ single mutant compared with that of controls and double mutants, the difference was not significant (ANOVA; df, 2; *F* = 0.5; *p* = 0.619) (Fig. 8*E*).

Our results show that, at E10.5, ventral OC morphology in the *Foxg1*^−/−^*;Wnt8b*^−/−^ double mutant resembles more that of *Foxg1*^−/−^*;Wnt8b*^+/+^ single mutants than that of controls, despite the fact that Pax6 expression is significantly reduced in these mutants compared with that of the *Foxg1*^−/−^*;Wnt8b*^+/+^ single mutant.

### Morphological and molecular alterations in OF development in *Foxg1*^−/−^ embryos are rescued in *Foxg1*^−/−^*;Wnt8b*^−/−^ double mutants by E11.5

**Figure 9.**
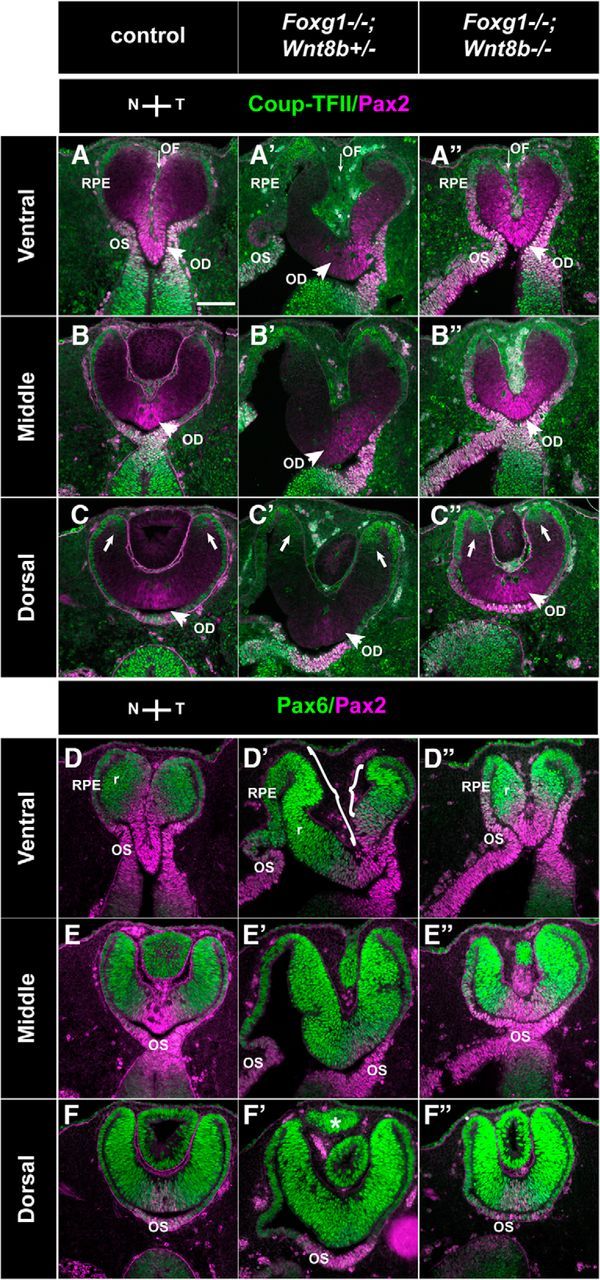
Marker analysis of the optic neuroepithelium in control, *Foxg1*^−/−^*;Wnt8b*^+/±^, and *Foxg1*^−/−^*;Wnt8b*^−/−^ E11.5 horizontal sections. Coup-TFII (green) and Pax2 (magenta) immunofluorescence in control (***A–C***), *Foxg1*^−/−^*;Wnt8b*^+/±^ (***A′–C′***), and *Foxg1*^−/−^*;Wnt8b*^−/−^ (***A″–C″***) sections. In ventral and middle sections, Coup-TFII is restricted in the RPE (***A–A″***, ***B–B″***), whereas in dorsal sections, it is expanded at the tips of the peripheral retina (***C–C″***, arrows). In ventral sections, Pax2 is detected at the apposed edges of the OF in controls (***A***) and *Foxg1*^−/−^*;Wnt8b*^−/−^ double mutants (***A″***). In *Foxg1*^−/−^*;Wnt8b*^+/±^ mutants, Pax2 expression is found in the temporal, but not the nasal, edges of the OF (***A′***). Pax2 is also detected in the optic disc (OD) marked with arrowheads in all three different genotypes (***A–A″***, ***B–B″***, ***C–C″***) and OS (***A–A″***, ***D–D″***, ***E–E″***). ***A–A″***, Thin arrows indicate the OF. Pax6 (green) and Pax2 (magenta) immunofluorescence in control (***D–F***), *Foxg1*^−/−^*;Wnt8b*^+/±^ (***D′–F′***), and *Foxg1*^−/−^*;Wnt8b*^−/−^ (***D″–F″***) sections. Pax6 expression is found in the retina (r) and RPE throughout the ventrodorsal axis. Pax6 staining reveals that the size of nasal and temporal retina is similar in controls (***D–F***) and *Foxg1*^−/−^*;Wnt8b*^−/−^ double mutants (***D″–F″***). In *Foxg1*^−/−^*;Wnt8b*^+/±^ mutants, the size of the nasal retina is clearly enlarged compared with that of the temporal retina (compare size of bracketed areas in ***D′***). ***F′***, *Presence of abnormal ciliary margin tissue in the *Foxg1*^−/−^*;Wnt8b*^+/±^ mutant, as previously described (Fotaki et al., 2013). Scale bars, 100 μm.

We then examined the morphology and marker expression of the developing OC at E11.5, using the same markers as for E10.5 embryos (Coup-TFII/Pax2 and Pax6/Pax2) (Fig. 9). As for the E10.5 embryos, results described below were consistent for all specimens from the same experimental group (6 eyes from 3 different embryos for each experimental group).

At E11.5, OC morphology in *Foxg1*^−/−^*;Wnt8b*^+/±^ mutants was severely compromised in ventral and middle sections (Fig. 9*A′–E′*), whereas the appearance of dorsal OC sections resembled more that of controls (Fig. 9*C*,*C′*,*F*,*F′*). Coup-TFII expression revealed a thickened RPE in ventronasal sections in this mutant (Fig. 9*A′*). The gap between the nasal and temporal edges of the OF was much greater in *Foxg1*^−/−^*;Wnt8b*^+/±^ mutants compared with controls (compare the OF region indicated by an arrow in Fig. 9*A* and Fig. 9*A′*) and Pax2 expression along the adjoining sides was reduced temporally and was absent nasally (Fig. 9*A′*,*D′*). Similar to controls (Fig. 9*D–F*), Pax6 expression in *Foxg1*^−/−^*;Wnt8b*^+/±^ mutants was detected in the retina and RPE (Fig. 9*D′–F′*). However, in ventral sections, Pax6 expression revealed a greater nasal domain compared with the Pax6-positive temporal domain (compare the bracketed nasal and temporal retinae in Fig. 9*D′*).

In contrast, in *Foxg1*^−/−^*;Wnt8b*^−/−^ double mutants, the anatomy of the OC resembled that of controls along the ventrodorsal axis (Fig. 9*A″–F″*). In ventral sections, the OC displayed a clearly centrally placed OF between the juxtaposed nasal and temporal sides (Fig. 9*A″*,*D″*). Although the distance between the edges of the OF in the *Foxg1*^−/−^*;Wnt8b*^−/−^ double mutant was not as narrow as that of controls, it was greatly reduced compared with that of the *Foxg1*^−/−^*;Wnt8b*^+/±^ mutant (compare the OF region indicated by an arrow in Fig. 9*A″* to that in Fig. 9*A*,*A′*). The Coup-TFII staining revealed a normal RPE appearance ventronasally and not the thickening observed in *Foxg1*^−/−^*;Wnt8b*^+/±^ mutants (Fig. 9*A″*). In ventral sections, Pax2 expression in the OC was increased compared with *Foxg1*^−/−^*;Wnt8b*^+/±^ mutants and surrounded both the nasal and temporal edges of the *Foxg1*^−/−^*;Wnt8b*^−/−^ OF (Fig. 9*A″*,*D″*). Similar to controls, Pax6 expression was detected in the OC and RPE (Fig. 9*D″–F″*).

**Figure 10.**
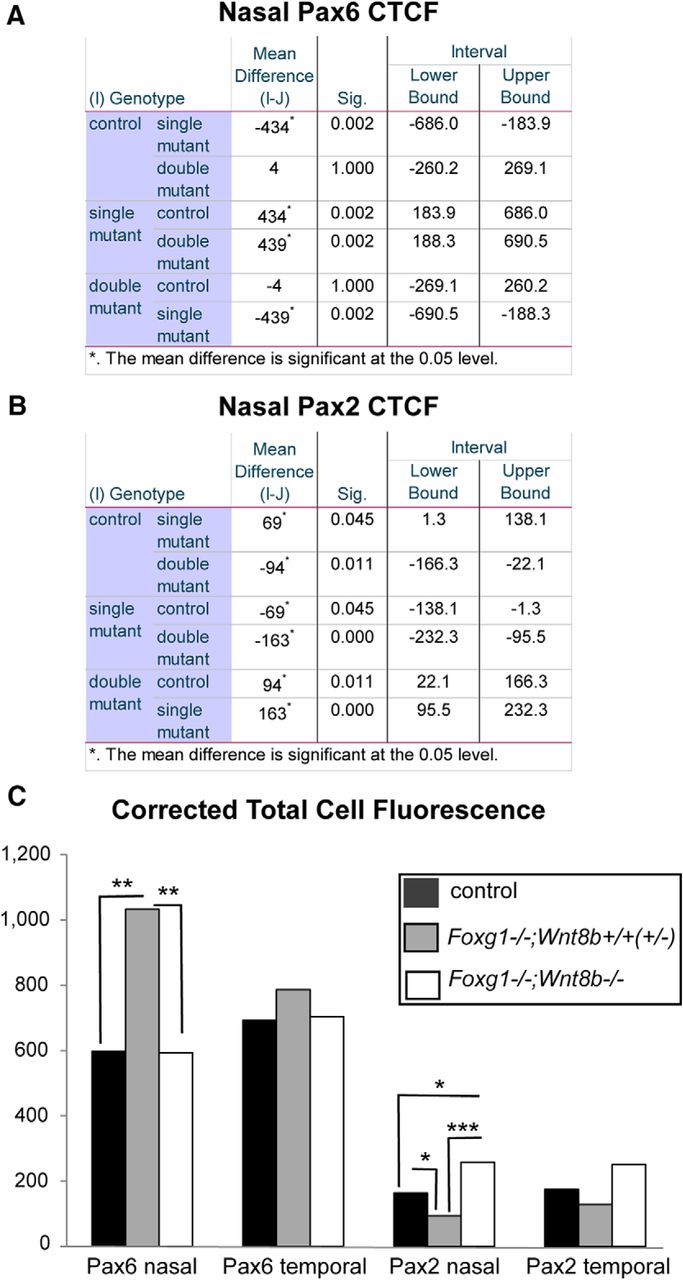
Quantitation of Pax6 and Pax2 expression in E11.5 retinal sections. An ANOVA was performed to define the statistical significance of the difference in the mean values of the CTCF for Pax6 (***A***) and Pax2 (***B***) in the nasal and temporal retinal domains along the ventrodorsal axis. ***C***, Graph represents these differences. Asterisks indicate corresponding *p* values. *n* = 4 eyes from 3 different embryos for all three groups analyzed.

Similar to E10.5, we quantitated expression of Pax6 and Pax2 using the corrected total cell fluorescence along the ventrodorsal axis. In agreement with our observations, we found a significant increase in Pax6 fluorescence (ANOVA; df, 2; *F* = 17.29; *p* = 0.001) (Fig. 10*A*,*C*) and a significant decrease in Pax2 fluorescence (ANOVA; df, 2; *F* = 23.665; *p* < 0.0001) (Fig. 10*B*,*C*) in *Foxg1*^−/−^*;Wnt8b*^+/±^ mutants compared with that of controls and double mutants. In addition, Pax2 fluorescence was significantly increased in double mutants compared with that of controls (Fig. 10*B*,*C*).

The above results show an amelioration of OC and OS morphology in the *Foxg1*^−/−^*;Wnt8b*^−/−^ double mutant by E11.5, with wild-type levels of OC Pax6 expression and a significant increase in nasal OC Pax2 expression compared with that of wild-types and *Foxg1*^−/−^*;Wnt8b*^+/±^ mutants.

**Figure 11.**
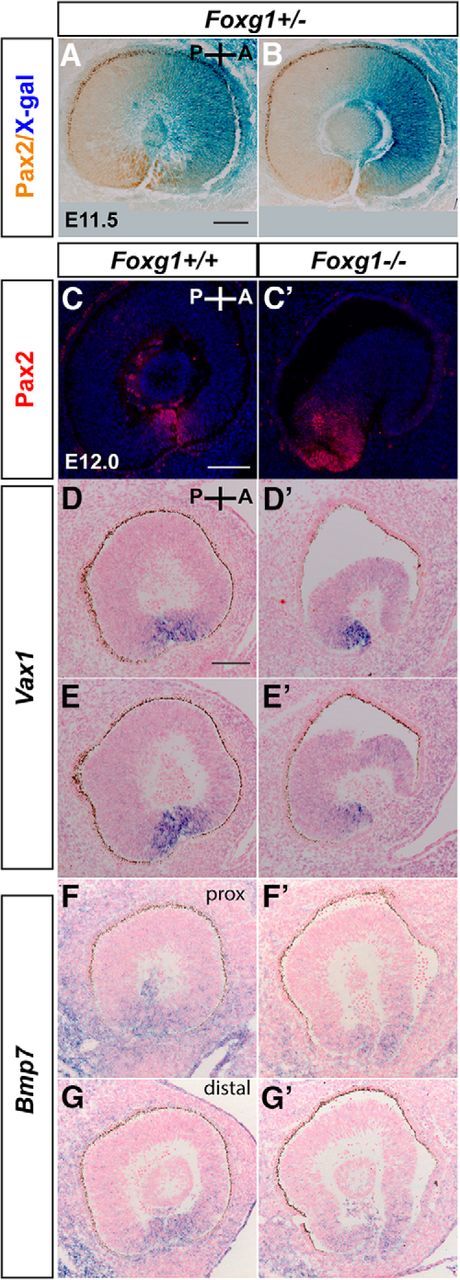
OF marker expression in control (*Foxg1*^+/+^; *Foxg1*^+/−^) and *Foxg1*^−/−^ mutant sagittal sections. At E11.5, Foxg1 (***A***, ***B***, blue staining) is expressed in the anterior (A) edge in controls (*Foxg1*^+/−^), whereas Pax2 (***A***, ***B***, brown staining) is detected in both the anterior and posterior (P) edges of the OF (***A***, ***B***). At E12.0, Pax2 immunofluorescence reveals normal anterior and posterior expression at the OF edges in the wild-type (*Foxg1*^+/+^) (***C***), whereas in the *Foxg1*^−/−^ mutant, the anterior domain of expression is lost and the posterior is maintained (***C′***). Similarly, *Vax1* mRNA wild-type anterior expression (***D***, ***E***) is compromised in the *Foxg1*^−/−^ mutant (***D′***, ***E′***), whereas posterior expression is intact (***D***, ***D′***, ***E***, ***E′***). *Bmp7* mRNA expression is intact in both the anterior and posterior edges of the OF in the *Foxg1*^−/−^ mutant (***F′***, ***G′***) similar to the wild-type (***F***, ***G***). ***A***, ***D***, ***D′***, ***F***, ***F′***, More proximal sections to ***B***, ***E***, ***E′***, ***G***, ***G′***, respectively. Scale bars: ***A***, ***B***, 100 μm; ***C***, ***C′***, 100 μm; ***D–G***, ***D′–G′***, 100 μm.

To validate rescue of Pax2 expression in the edges of the *Foxg1*^−/−^*;Wnt8b*^−/−^ OF, we further examined sagittal sections immunostained for Pax2. First, we examined Pax2 expression in *Foxg1*^−/−^ single mutants and wild-type littermates at E12.0, when OF closure is initiated in controls (Fig. 11). In contrast to wild-type expression (Fig. 11*A–C*), in *Foxg1*^−/−^ retinae, the anterior tip was Pax2-negative and located at a distance from the Pax2-positive-posterior OF tip (Fig. 11*C′*). Similar results were obtained with *in situ* hybridization for *Vax1* (Fig. 11*D*,*D′*,*E*,*E′*), which is also expressed at the edges of the OF (Bäumer et al., 2002) and, when mutated, gives rise to a coloboma phenotype in mice (Hallonet et al., 1999). However, similar expression of *Bmp7*, a gene essential for OF formation (Morcillo et al., 2006), was observed in both the anterior and posterior tips of wild-types and *Foxg1*^−/−^ mutants along the proximodistal axis (Fig. 11*F*,*F′*,*G*,*G′*).

**Figure 12.**
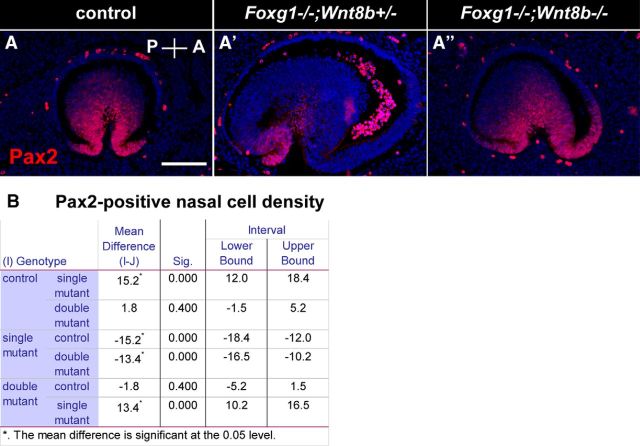
Pax2 expression in the anterior and posterior tips of the OF. Pax2 expression in E11.5 sagittal control (***A***), *Foxg1*^−/−^*;Wnt8b*^+/±^ mutant (***A′***), and *Foxg1*^−/−^*;Wnt8b*^−/−^ double mutant (***A″***) sections showing that the normal Pax2 expression in the anterior (A) and posterior (P) edges of the control OF (***A***) is only found posteriorly in the *Foxg1*^−/−^*;Wnt8b*^+/±^ mutant (***A′***) but is rescued in the *Foxg1*^−/−^*;Wnt8b*^−/−^ double mutant (***A″***). ANOVA revealed that the Pax2-positive cells within a 0.01 mm^2^ area at the edges of the nasal retina is significantly reduced (*p* < 0.0001) in the *Foxg1*^−/−^*;Wnt8b*^+/±^ mutants compared with that of controls and double mutants (***B***). *n*^control^ = 3 eyes from 3 different embryos; *n*^*Foxg1*−/−*;Wnt8b*+/±^ = 4 eyes from 3 different embryos; *n*^*Foxg1*−/−*;Wnt8b*−/−^ = 3 eyes from 3 different embryos. Scale bars, 100 μm.

In *Foxg1*^−/−^*;Wnt8b*^−/−^ E11.5 sagittal sections (4 eyes from 3 different embryos in the sagittal plane), Pax2 expression was present in both the anterior and posterior tips of the *Foxg1*^−/−^*;Wnt8b*^−/−^ OF (Fig. 12*A″*) along the proximodistal axis (3 of 4 eyes examined), similar to controls (Fig. 12*A*). In one case, anterior Pax2 staining in distal sections was limited to only a few cells (data not shown). However, in the *Foxg1*^−/−^*;Wnt8b*^+/±^ mutant, Pax2 expression was not detected in the anterior OF (Fig. 12*A′*), in agreement with the results we observed in the *Foxg1*^−/−^ single mutant (Fig. 11*C*,*C′*). This was further confirmed by counting the Pax2-positive cells at the edges of the OF within a square area of 0.01 mm^2^ (for details, see Materials and Methods). The Pax2-positive cell density in the nasal edge of the OF was significantly reduced in the *Foxg1*^−/−^*;Wnt8b*^+/±^ mutant compared with that of controls and double mutants (ANOVA; df, 2; *F* = 141,374; *p* < 0.0001) (Fig. 12*B*). No differences were observed in temporal Pax2-positive cell density between groups (ANOVA; df, 2; *F* = 3,669; *p* = 0.081).

These results confirm that loss of Wnt8b function in the *Foxg1*^−/−^ null background leads to a specific increase in Pax2 expression in the anterior (nasal) tips of the OF, strongly suggesting that this may contribute to the rescue of the coloboma phenotype in the *Foxg1*^−/−^*;Wnt8b*^−/−^ double mutant.

### Cell proliferation is not altered in the *Foxg1*^−/−^ mutant at E10.5

OC formation and OF closure rely on balanced cell proliferation and cell death (Morcillo et al., 2006; See and Clagett-Dame, 2009; Cai et al., 2013; Noh et al., 2016).

**Figure 13.**
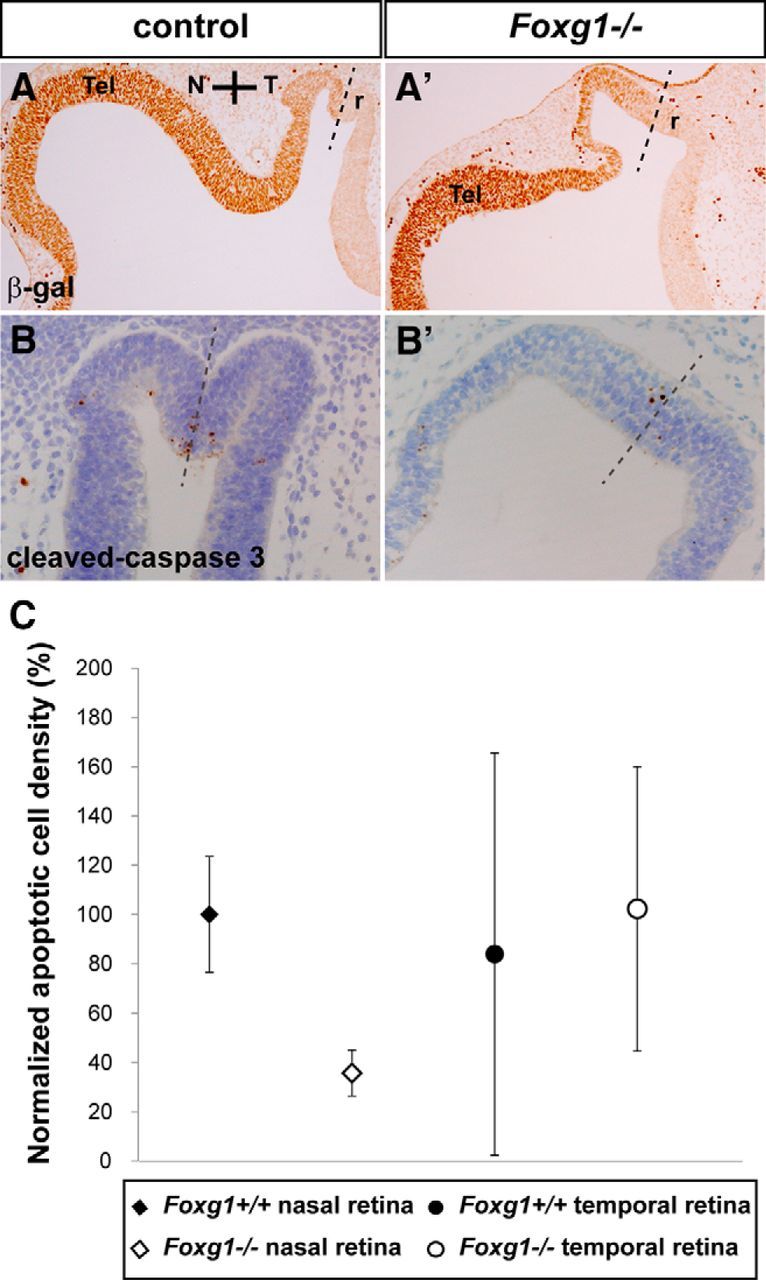
Apoptotic cell density is significantly reduced in the nasal retina in the *Foxg1*^−/−^ mutant. β-gal staining defines the border between the nasal and temporal retinae (r) (***A***, ***A′***, dashed line) in *Foxg1*^+/−^ heterozygote controls (***A***) and *Foxg1*^−/−^ mutants (***A′***). Cleaved caspase-3 immunohistochemistry was used to detect retinal cells undergoing apoptotic cell death in wild-types (*Foxg1*^+/+^) (***B***) and *Foxg1*^−/−^ mutants (***B′***). Mean apoptotic cell density values were normalized as a percentage to the wild-type nasal value (100% apoptotic density) (***C***). Scale bars, 50 μm.

To understand whether aberrant OC cell proliferation may be involved in coloboma formation in the *Foxg1*^−/−^ mutant, we calculated the LI (BrDU-positive cells over total number of cells) in nasal and temporal retinae of E10.5 wild-type and *Foxg1*^−/−^ mutant OCs (for a detailed description, see Materials and Methods). To define the border between nasal and temporal retina, we used as a guide *Foxg1*^+/−^ heterozygous and *Foxg1*^−/−^ homozygous sections immunostained for β-galactosidase (β-gal) (Xuan et al., 1995), normally found in the nasal retina (Fig. 13*A*,*A′*, dashed line). Our cell counts did not reveal any significant differences in the LI ± SE among wild-type (0.45170 ± 0.05608) and *Foxg1*^−/−^ mutant (0.45398 ± 0.03902) nasal retina or wild-type (0.42959 ± 0.03825) and *Foxg1*^−/−^ mutant (0.43340 ± 0.05842) temporal retina. In addition, immunohistochemistry for phosphorylated histone H3 (pHH3), a mitosis (M)-phase marker (Hans and Dimitrov, 2001), did not result in any differences in the cell distribution or the mitotic index (number of pHH3-positive cells per surface area ± SE) (for details, see Materials and Methods) between E10.5 wild-type (0.03483 ± 0.00454) and *Foxg1*^−/−^ mutant (0.03418 ± 0.00266) nasal retina or wild-type (0.03133 ± 0.00337) and *Foxg1*^−/−^ mutant (0.03686 ± 0.00772) temporal retina. Finally, based on the suggestion that precocious differentiation of neural progenitors at the apposed edges of the OF may result in failure of the fissure to seal (Lohnes et al., 1994), we performed immunohistochemistry for β-tubulin III (Tuj1), a marker of early born neurons (Memberg and Hall, 1995). However, we did not observe any Tuj1-positive cells in the E10.5 developing OC or OS of wild-types or *Foxg1*^−/−^ mutants (data not shown).

### Apoptotic defects in the nasal retina in the *Foxg1*^−/−^ mutant at E10.5 are partially rescued in the *Foxg1*^−/−^*;Wnt8b*^−/−^ double mutant

To determine whether abnormal apoptosis is associated with the OC phenotype of *Foxg1*^−/−^ mutants, we examined the apoptotic density in E10.5 nasal and temporal wild-type and *Foxg1*^−/−^ horizontal sections, using immunohistochemistry for cleaved caspase-3 (Noh et al., 2016). Our cell counts revealed a significantly lower density of apoptotic cells (*p* < 0.05) in the *Foxg1*^−/−^ nasal retina compared with that of wild-types (Fig. 13; Table 2), with a mean value reaching 35 pp (percentage points) of the wild-type nasal values. No differences in apoptotic density were observed among genotypes in the temporal retina (Fig. 13; Table 2).

**Figure 14.**
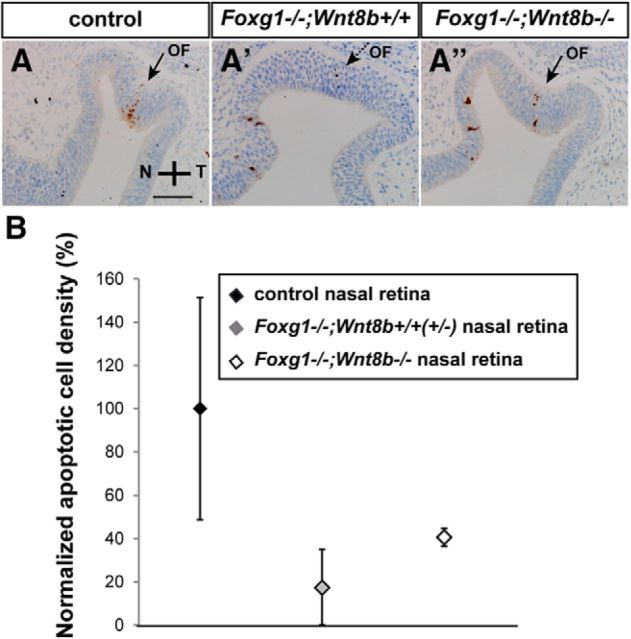
Partial rescue of the apoptosis phenotype of the *Foxg1*^−/−^ mutant in the *Foxg1*^−/−^*;Wnt8b*^−/−^ double mutant. Cleaved caspase-3 immunohistochemistry labels retinal cells undergoing apoptotic cell death in control (***A***), *Foxg1*^−/−^*;Wnt8b*^+/±^ (***A′***), and *Foxg1*^−/−^*;Wnt8b*^−/−^ (***A″***) E10.5 horizontal sections. ***A***, ***A″***, Arrows indicate the OF, which starts to form in the control and *Foxg1*^−/−^*;Wnt8b*^−/−^ double mutant. ***A′***, Dashed arrow indicates a less-clear formation of the OF in *Foxg1*^−/−^*;Wnt8b*^+/+^single mutants. Mean apoptotic cell density values were normalized as a percentage to the control nasal value (100% apoptotic density) (***B***). Scale bars, 50 μm.

The correlation between decreased nasal apoptosis and failed OF closure in *Foxg1*^−/−^ mutants led us to hypothesize that the rescue of OF closure in the *Foxg1*^−/−^*;Wnt8b*^−/−^ double mutant observed at E11.5 (Fig. 9*A″*,*D″*) is associated with restoration of normal apoptotic levels. We analyzed apoptotic cell density in three experimental groups: (1) controls, (2) *Foxg1*^−/−^*;Wnt8b*^+/±^ mutants, and (3) *Foxg1*^−/−^*;Wnt8b*^−/−^ double mutants. In nasal retina, and in accordance with our hypothesis, we found an increase of 24pp in mean apoptotic density in *Foxg1*^−/−^*;Wnt8b*^−/−^ double mutants compared with *Foxg1*^−/−^*;Wnt8b*^+/±^ mutants, which was statistically significant (*p* < 0.05) (Fig. 14; Table 3), although the increase did not quite reach the level observed in our control samples (*p* < 0.05) (Fig. 14; Table 3). No significant differences were observed in temporal apoptotic cell density between controls, *Foxg1*^−/−^*;Wnt8b*^+/±^ mutants, and *Foxg1*^−/−^*;Wnt8b*^−/−^ double mutants (Table 3).

Our results show that, at E10.5, there is significant increase in nasal apoptotic cell density in the *Foxg1*^−/−^*;Wnt8b*^−/−^ double mutant compared with the *Foxg1*^−/−^*;Wnt8b*^+/±^ mutant, consistent with the idea that this may be partly responsible for the amelioration of the *Foxg1*^−/−^*;Wnt8b*^−/−^ OF morphology at E11.5.

### Upregulation of Wnt/β-catenin signaling target molecules in the *Foxg1*^−/−^ OC and OS

Wnt molecules signal mainly through the Wnt/β-catenin or the planar cell polarity pathways (for review, see Loh et al., 2016). To gain insight into which Wnt signaling pathway is affected by the upregulation of *Wnt8b* observed in the OS of the *Foxg1*^−/−^ mutant, we examined downstream targets of the Wnt/β-catenin and the planar cell polarity pathways in the OC and OS.

**Figure 15.**
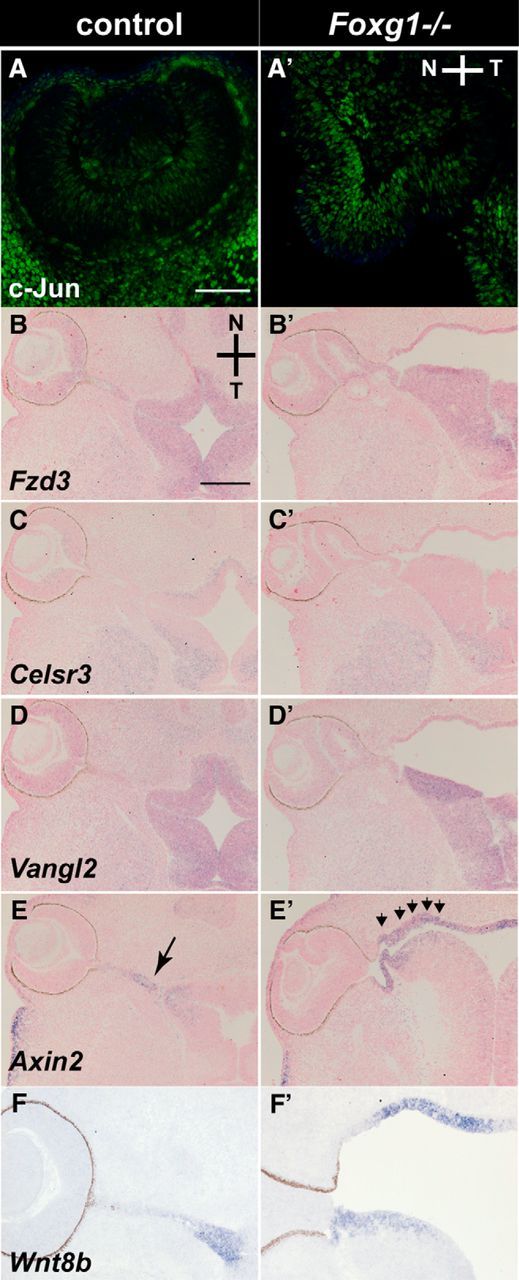
OC and OS Wnt-signaling target gene analysis in controls and *Foxg1*^−/−^ mutants at E12.5. c-Jun protein expression is upregulated in the *Foxg1*^−/−^ OC (***A′***) compared with expression in controls (***A***). No differences in *Fzd3* (***B***, ***B′***), *Celsr3* (***C***, ***C′***), or *Vangl2* (***D***, ***D′)*** mRNA expression are observed between *Foxg1*^−/−^ mutants and controls. *Axin2* expression is upregulated in the nasal component of the OS in *Foxg1*^−/−^ mutants (***E′***, arrowheads) compared with controls (***E***, arrow) and mirrors upregulated expression of *Wnt8b* in the same region (compare ***E*** with ***F*** and ***E′*** with ***F′***). Scale bars: ***A***, ***A′***, 100 μm; ***B–E***, ***B′-E′***, 200 μm; ***F***, ***F′***, 100 μm.

First, we performed a PCR array analysis and profiled the expression of 84 genes related to Wnt-mediated signal transduction, using RNA extracted from OCs of E11.0 wild-type and *Foxg1*^−/−^ embryos. Of 12 Wnt-signaling target genes included in the array (Table 4, asterisk), the only one that showed a significant change in expression was c-Jun, a downstream target of the Wnt/β-catenin pathway (Mann et al., 1999), with a 2.5-fold upregulation (*p* = 0.01) in *Foxg1*^−/−^ OCs (Table 4). This upregulation became apparent at the protein level by E12.0, when we detected a clear increase in c-Jun expression in the *Foxg1*^−/−^ retina (Fig. 15*A*,*A′*) (results were consistent in *n* = 6 from 3 different control and *Foxg1*^−/−^ mutant eyes).

**Table 4.** PCR array results for 84 genes related to Wnt-mediated signal transduction, showing the average ΔC_t_ values for each experimental group, fold changes, and the *p* values of these changes*^a^*

Gene symbol	AVG ΔC_t_		Fold change	*p*
	*Foxg1*^−/−^	*Foxg1*^+/+^		
*Aes*	3.34	3.43	1.06	0.922448
*Apc*	6.5	6.67	1.13	0.844910
*Axin1*	5.95	5.98	1.02	0.986177
Axin2*	4.96	5.56	1.52	0.282091
*Bcl9*	5.93	6.26	1.25	0.789822
Btrc*	6.09	6.11	1.01	0.955878
Ccnd1*	3.2	2.87	0.8	0.446753
Ccnd2*	3.46	3.22	0.84	0.561827
*Csnk1a1*	3.31	3.1	0.86	0.629207
*Csnk2a1*	4.64	4.48	0.9	0.725660
*Ctbp1*	4.51	4.52	1.01	0.868870
*Ctnnb1*	2.24	2.2	0.97	0.966749
*Ctnnbip1*	6.15	6.49	1.27	0.523909
*Daam1*	8.01	8.25	1.18	0.952898
Dab2*	5.77	6.55	1.71	0.083046
*Dixdc1*	5.83	6.15	1.25	0.601836
*Dkk1*	7.4	7.22	0.88	0.575939
*Dkk3*	4.65	4.66	1.01	0.957113
*Dvl1*	6.78	6.88	1.07	0.677430
*Dvl2*	5.9	5.94	1.03	0.961346
*Ep300*	6.54	6.2	0.79	0.681840
*Fbxw11*	5.43	5.32	0.93	0.738350
*Fbxw4*	7.39	7.37	0.99	0.989112
*Fgf4*	Undetermined	Undetermined	—	—
Fosl1*	15	14.2	0.57	0.462573
*Foxn1*	Undetermined	Undetermined	—	—
*Frat1*	9.02	9.17	1.11	0.633178
*Frzb*	7.61	9.07	2.74	0.175782
*Fzd1*	4.58	5.08	1.42	0.306249
*Fzd2*	5.28	5.89	1.52	0.330346
*Fzd3*	4.95	4.99	1.03	0.939483
*Fzd4*	5.92	6.28	1.29	0.565771
*Fzd5*	5.28	4.44	0.56	0.201944
*Fzd6*	7.42	7.67	1.19	0.505062
*Fzd7*	4.69	5.07	1.31	0.382821
*Fzd8*	8.89	9.24	1.27	0.165558
*Fzd9*	12.09	12.67	1.5	0.267910
*Gsk3b*	4.47	4.65	1.13	0.810280
Jun*	4.42	5.74	2.5	0.019267
*Kremen1*	6.88	7.22	1.27	0.575288
*Lef1*	5.7	6.33	1.55	0.137805
*Lrp5*	5.41	5.71	1.23	0.340422
*Lrp6*	4.2	4.52	1.24	0.485828
*Mapk8*	5.23	5.45	1.16	0.485842
Mmp7*	Undetermined	Undetermined	—	—
Myc*	6.1	6.12	1.02	0.912680
*Nfatc1*	8.78	9.39	1.52	0.340969
*Nkd1*	4.89	5.23	1.27	0.215351
*Nlk*	6.17	5.74	0.75	0.284165
Pitx2*	7.29	7.52	1.17	0.668072
*Porcn*	7.98	7.94	0.97	0.729696
Ppard*	7.86	7.69	0.89	0.807290
*Prickle1*	7.02	7.76	1.67	0.173155
*Pygo1*	6.79	7.37	1.5	0.190255
*Rhoa*	3.12	2.82	0.81	0.581906
*Rhou*	7.2	7.16	0.97	0.932350
*Ruvbl1*	4.36	4	0.78	0.368340
*Sfrp1*	4.74	4.31	0.74	0.218370
*Sfrp2*	1.58	1.06	0.7	0.049288
*Sfrp4*	Undetermined	Undetermined	—	—
*Sox17*	11.66	11.61	0.96	0.922776
*Tcf7*	7.69	7.53	0.9	0.751242
*Tcf7l1*	6.68	6.76	1.06	0.762543
*Tle1*	6.6	6.7	1.07	0.725797
*Vangl2*	5	5.57	1.49	0.081913
*Wif1*	8.34	8.95	1.53	0.360760
Wisp1*	11.52	12.38	1.82	0.415085
*Wnt1*	16.13	15.58	0.68	0.402762
*Wnt10a*	12.98	14.27	2.44	0.114037
*Wnt11*	9.62	11.86	4.73	0.042069
*Wnt16*	10.32	11.6	2.43	0.084045
*Wnt2*	Undetermined	Undetermined	—	—
*Wnt2b*	6.75	6.77	1.02	0.973415
*Wnt3*	13.7	14.88	2.26	0.005500
*Wnt3a*	13.97	14.96	1.99	0.206819
*Wnt4*	8.57	10.26	3.21	0.004796
*Wnt5a*	5.68	6.84	2.23	0.095104
*Wnt5b*	6.58	6.56	0.98	0.910277
*Wnt6*	7.84	8.8	1.95	0.065652
*Wnt7a*	12.37	12.01	0.78	0.438452
*Wnt7b*	9.21	9.17	0.97	0.870328
*Wnt8a*	Undetermined	Undetermined	—	—
*Wnt8b*	9.14	10.08	1.92	0.371946
*Wnt9a*	Undetermined	Undetermined	—	—

*^a^*RNA was extracted from optic cups at E11.0. Results are the average values from three control and three mutant plates. Fold changes >2 with a *p* value <0.05 were observed for *c-Jun*, *Wnt11*, *Wnt3*, and *Wnt4*.

*Downstream targets of the Wnt signaling pathway.

We then analyzed mRNA expression by means of *in situ* hybridization in the OS of wild-type and *Foxg1*^−/−^ mutants of molecules implicated in the planar cell polarity pathway (*Fzd3*, *Celsr3*, and *Vangl3*) (Fig. 15*B–D*,*B′–D′*) (Tissir et al., 2005; Montcouquiol et al., 2006), as well as *Axin2* (Fig. 15*E*,*E′*), a read-out of the Wnt/β-catenin signaling pathway (Jho et al., 2002). *Axin2* showed a clear upregulation in the OS of the *Foxg1*^−/−^ mutant (Fig. 15*E′*), similar to the upregulation observed in *Wnt8b* expression (Fig. 15*F*,*F′*), which became evident as early as E10.5 (data not shown).

These results show that Wn8b upregulation in the *Foxg1*^−/−^ OS results in upregulation of the Wnt/β-catenin signaling pathway through overexpression of the downstream targets *c-Jun* and *Axin2* in the *Foxg1*^−/−^ mutant OC and OS, respectively.

## Discussion

**Figure 16.**
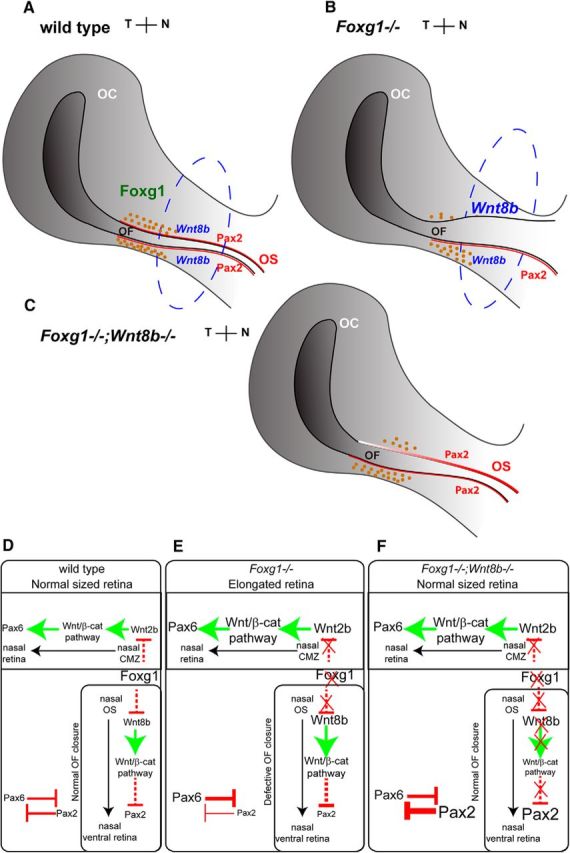
Schematic summary of main findings and proposed Foxg1 regulatory network in the developing eye. ***A***, In wild-types, Foxg1 is normally expressed in the nasal OC and OS, *Wnt8b* is expressed in the nasal and temporal OS, and Pax2 is expressed in the nasal and temporal OS and in the nasal and temporal edges of the OF. Apoptosis is normally observed in the nasal and temporal edges of the OF (brown dots). ***B***, In *Foxg1*^−/−^ mutants, loss of Foxg1 results in abnormal upregulation of *Wnt8b* nasally, increased suppression of Pax2, and decreased apoptotic cell death in the nasal edges of the fissure. ***C***, In *Foxg1*^−/−^*;Wnt8b*^−/−^ double mutants, loss of both Foxg1 and Wnt8b results in rescued Pax2 expression in the nasal edges of the OF, although Pax2 expression is stronger distally than proximally. Apoptosis is increased compared with the *Foxg1*^−/−^ mutant (***B***), but it is still significantly below the control values (***A***). ***D***–***F***, A model of possible molecular interactions showing how Foxg1 function and loss of function affects OC and OF formation. —|, Repression; ->, activation. Our model cannot predict whether the molecular interactions are direct or indirect.

Our data unravel a novel mechanism of OF closure, which relies on Foxg1-mediated suppression of *Wnt8b* in the nasal OS, resulting in balanced apoptosis and normal Pax2 expression in the nasal edges of the fissure (Fig. 16*A–C*). This newly described role of Foxg1 in OC formation is independent of its function as a retinal nasotemporal determinant.

We hypothesized that, similar to foxg1 function in the zebrafish telencephalon (Danesin et al., 2009), mouse Foxg1 suppresses Wnt8b function in the developing optic neuroepithelium for proper OC formation to take place. Indeed, wild-type *Wnt8b* expression in the OS was upregulated in the *Foxg1*^−/−^ mutant as early as E10.5. This was observed in the nasal stalk, which is the region of the developing optic neuroepithelium with highest *Foxg1* expression levels at this developmental stage (Hatini et al., 1994; current study). Our genetic experiment allowed us to evaluate the *in vivo* effects of loss of Wnt8b expression in a *Foxg1*-null genetic background. The remarkable rescue of the OC and OS morphology and the substantial sealing of the OF by E15.5 in the *Foxg1*^−/−^*;Wnt8b*^−/−^ mutant are in agreement with our hypothesis. Future experiments will show whether mouse retinal Foxg1 suppresses Wnt8b by direct binding, similar to zebrafish telencephalon (Danesin et al., 2009), or whether the suppression is indirect, supported by the lack of Foxg1 binding sites within the Wnt8b promoter region in mouse neural stem cells (Bulstrode et al., 2017).

Foxg1 is expressed in the nasal retina of vertebrates (Hatini et al., 1994; Takahashi et al., 2003; Picker et al., 2009), and work in chick and zebrafish has established Foxg1's role as an early determinant of retinal nasotemporal polarity exerting an antagonistic effect on Foxd1 (Yuasa et al., 1996; Takahashi et al., 2003), a temporal retina determinant that is abnormally expressed in the *Foxg1*^−/−^ nasal retina (Huh et al., 1999; Tian et al., 2008). The fact that abnormal Foxd1 expression in the nasal retina is still observed in the *Foxg1*^−/−^*;Wnt8b*^−/−^ mutant reveals that the coloboma phenotype is not a secondary defect to the abnormal *Foxg1*^−/−^ nasotemporal patterning.

Although the shape of the *Foxg1*^−/−^*;Wnt8b*^−/−^ OC resembles that of the *Foxg1*^−/−^ mutant at E10.5, its Pax6 and Pax2 expression profiles mimic those of controls. By E11.5, the *Foxg1*^−/−^*;Wnt8b*^−/−^ OC morphology resembles that of controls, with normal Pax6 and elevated Pax2 expression levels. The OC normally undergoes a series of morphological changes, from a flattened to a spherical shape (Lamb et al., 2007; Eiraku et al., 2011; Eiraku and Sasai, 2012). Our data support the idea that, in the *Foxg1*^−/−^*;Wnt8b*^−/−^ mutant, OC formation is delayed, resulting in closure of the OF at a later developmental point, at ∼E15.5 rather than at E13.5 (Hero, 1989).

Pax2 is required for OF closure (Torres et al., 1996), and loss of Pax2 expression in the nasal (anterior) edge of the OF in the *Foxg1*^−/−^ mutant may account for failure of the OF edges to fuse. This is further supported by the fact that, in the *Foxg1*^−/−^*;Wnt8b*^−/−^ mutant with 100% OF fusion in proximal sections, there is 100% rescue of Pax2 expression in the anterior OF proximally, whereas in distal sections, where Pax2 expression is not fully recovered, we observe less efficient rescue (Fig. 16*A–C*).

Precise regulation of cellular events is crucial for the development of the OC and OS. At early stages of OC development, intense cell proliferation takes place, which is associated with the invagination of the optic vesicle and the appearance of the OF (Calvente et al., 1988). We hypothesized that changes in OC morphology in the *Foxg1*^−/−^ mutant may result from aberrant proliferation, which may be rescued in the *Foxg1*^−/−^*;Wnt8b*^−/−^ mutant. However, the lack of difference in the LI, mitotic index, and β-tubulin-III expression between wild-types and *Foxg1*^−/−^ mutants at E10.5 argued against the idea that aberrant proliferation and/or premature differentiation is involved in the early *Foxg1*^−/−^ OC morphological defects.

Programmed cell death (apoptosis) in the nasal and temporal edges adjoining the OF normally occurs during mouse OC formation and OF closure (Hero, 1989; Ozeki et al., 2000). Apoptosis is first detected in ventronasal retina in the region of the presumptive OF at E9.5 and is then found in the OF edges, becoming undetectable after the edges fuse at E13.5 (Ozeki et al., 2000). In addition, either increased or decreased OF apoptosis has been found in mouse mutants with a coloboma phenotype (Cai et al., 2013; Noh et al., 2016). This evidence strongly supports balanced apoptosis at the edges of the OF as a major determinant of proper OF closure. Our data show significantly reduced levels of caspase-3-mediated apoptosis in the *Foxg1*^−/−^ ventronasal retina, which strongly suggest a requirement for Foxg1 in promoting apoptotic cell death at the nasal edge of the fissure. Reduction in apoptosis is observed in *Bmp7*^−/−^ mouse mutants, which fail to form OF (Morcillo et al., 2006). However, normal *Bmp7* expression in the edges of the *Foxg1*^−/−^ fissure indicates that Foxg1 OF function is independent of Bmp7. In the *Foxg1*^−/−^*;Wnt8b*^−/−^ double mutant, apoptosis in the nasal retina was significantly higher to that of *Foxg1*^−/−^*;Wnt8b*^+/±^ mutants, suggesting that Wnt8b overexpression is a contributing factor to the reduction in apoptosis we observe in the *Foxg1*^−/−^ embryos.

Wnt8b activates Wnt/β-catenin signaling (Lee et al., 2006), and our data suggest that upregulation of this signaling cascade results in coloboma. Although our present data do not provide a direct link between the observed changes in apoptosis and Wnt/β-catenin signaling, it is interesting that the only downstream target with upregulated expression in the *Foxg1*^−/−^ OC was *c-Jun*. c-Jun protects cells from excessive apoptotic activity (Wisdom et al., 1999; Shaulian and Karin, 2002), and its upregulation in the *Foxg1*^−/−^ mutant OC may result in reduction of apoptosis in the nasal edge of the OF compromising fissure closure.

Although our array analysis in the OC did not reveal differences between wild-types and *Foxg1*^−/−^ mutants in *Axin2* expression, a read-out of the Wnt/β-catenin pathway (Jho et al., 2002), *Axin2* was found upregulated in the *Foxg1*^−/−^ OS, at the same sites where *Wnt8b* upregulation was observed. This is in agreement with the current notion that Wnt proteins are locally acting signaling molecules (Alexandre et al., 2014; Farin et al., 2016; Loh et al., 2016) and suggests that the rescued OC morphology in the *Foxg1*^−/−^*;Wnt8b*^−/−^ mutant may be a secondary effect to a primary rescue in OS formation. This idea is further supported with the following model of molecular interactions in the developing eye at ∼E11.5 (Fig. 16*D–F*).

Foxg1 in the nasal ciliary margin controls Wnt2b levels, which in turn result in normal transcriptional activation of Pax6. In parallel, Foxg1 in the nasal stalk keeps Wnt8b levels in check, resulting in normal Pax2 expression in the ventral retina, due to low transcriptional repression. Finally, Pax6 and Pax2 levels are balanced through reciprocal inhibition, as previously described (Schwarz et al., 2000) (Fig. 16*D*). When Foxg1 function is abolished, Pax6 expression is upregulated, resulting in an expanded ciliary margin (Fotaki et al., 2013), whereas Pax2 expression is significantly reduced (this study), resulting in failure of the OF to form properly (Fig. 16*E*). In the *Foxg1*^−/−^*;Wnt8b*^−/−^ mutant, Pax2 levels are significantly increased compared with those of single mutants and controls (this study), resulting in proper OF closure. Although Pax6 expression should still be elevated in the double mutant, our quantitation analysis reveals similar levels of expression to those of controls. A possible explanation for this is that, in the double mutant, Pax2 expression is more elevated than that of Pax6; and when it suppresses Pax6, it reduces it to normal values (Fig. 16*F*). Although currently unavailable, we predict that a *Pax2*-overexpressing mouse strain crossed to the *Foxg1*-mutant background will phenocopy the *Foxg1*^−/−^*;Wnt8b*^−/−^ phenotype in line with our hypothesis.

Our observations that upregulated Wnt/β-catenin signaling associates with coloboma formation seem to be at odds with the fact that a coloboma phenotype is also observed in cases when the Wnt/β-catenin signaling is reduced, as in the case of humans with mutations in the Wnt receptor gene *FZD5* (Liu et al., 2016) and *Fzd5*^−/−^ null mice, which show increased apoptosis and increased Pax2 expression in the OC (Liu and Nathans, 2008). However, Wnt/β-catenin signaling must also be increased in *Dkk1*^+/−^ mice with reduced levels of the Wnt antagonist Dkk1 and in mice with a loss of function of Axin2, a negative regulator of the Wnt/β-catenin signaling, both of which also show a coloboma phenotype (Lieven and Rüther, 2011; Alldredge and Fuhrmann, 2016). This all suggests that Wnt/β-catenin signaling needs to be tightly regulated in the OC and OS for proper OF closure, and unbalanced expression (overexpression or underexpression) of its components leads to coloboma.

Our work uncovers a novel action of Foxg1 in limiting Wnt/β-catenin signaling in the OS for proper OC and OS formation and OF closure to take place and provides additional knowledge regarding the molecular players and cellular mechanisms underlying coloboma formation.
